# Lymphocyte deficiency limits Epstein-Barr virus latent membrane protein 1 induced chronic inflammation and carcinogenic pathology *in vivo*

**DOI:** 10.1186/1476-4598-10-11

**Published:** 2011-02-03

**Authors:** Adele Hannigan, Asif M Qureshi, Colin Nixon, Penelope M Tsimbouri, Sarah Jones, Adrian W Philbey, Joanna B Wilson

**Affiliations:** 1 College of Medical, Veterinary & Life Sciences, University of Glasgow, Glasgow G12 8QQ, UK; 2 Work in this paper was conducted while at address 1, current address: Engeneic Ltd, Building 2, 25 Sirius Road, Lane Cove West, NSW 2066, Australia; 3 Beatson Institute for Cancer Research, Bearsden, Glasgow G61 1BD, UK

## Abstract

**Background:**

The importance of the malignant cell environment to its growth and survival is becoming increasingly apparent, with dynamic cross talk between the neoplastic cell, the leukocyte infiltrate and the stroma. Most cancers are accompanied by leukocyte infiltration which, contrary to an anticipated immuno-protective role, could be contributing to tumour development and cancer progression. Epstein-Barr virus (EBV) associated cancers, including nasopharyngeal carcinoma and Hodgkin's Disease, show a considerable leukocyte infiltration which surrounds the neoplastic cells, raising the questions as to what role these cells play in either restricting or supporting the tumour and what draws the cells into the tumour. In order to begin to address this we have studied a transgenic model of multistage carcinogenesis with epithelial expression of the EBV primary oncoprotein, latent membrane protein 1 (LMP1). LMP1 is expressed particularly in the skin, which develops a hyperplastic pathology soon after birth.

**Results:**

The pathology advances with time leading to erosive dermatitis which is inflamed with a mixed infiltrate involving activated CD8+ T-cells, CD4+ T-cells including CD4+/CD25+/FoxP3+ Treg cells, mast cells and neutrophils. Also significant dermal deposition of immunoglobulin-G (IgG) is observed as the pathology advances. Along with NF-kappaB activation, STAT3, a central factor in inflammation regulation, is activated in the transgenic tissue. Several inflammatory factors are subsequently upregulated, notably CD30 and its ligand CD153, also leukocyte trafficking factors including CXCL10, CXCL13, L-selectin and TGFβ1, and inflammatory cytokines including IL-1β, IL-3 and the murine IL-8 analogues CXCL1, CXCL2 and CXCL5-6, amongst others. The crucial role of mature T- and/or B-lymphocytes in the advancing pathology is demonstrated by their elimination, which precludes mast cell infiltration and limits the pathology to an early, benign stage.

**Conclusions:**

LMP1 can lead to the activation of several key factors mediating proliferation, angiogenesis and inflammation *in vivo*. With the initiation of an inflammatory programme, leukocyte recruitment follows which then itself contributes to the progressing pathology in these transgenic mice, with a pivotal role for B-and/or T-cells in the process. The model suggests a basis for the leukocyte infiltrate observed in EBV-associated cancer and its supporting role, as well as potential points for therapeutic intervention.

## Background

There is an increasing body of evidence linking chronic inflammation and cancer, the complexities of which are beginning to be unravelled. Inflammation is characterised by the influx of immune cells to a localised site where they release and respond to factors in a dynamic state. Under normal circumstances, this occurs to promote wound repair and combat infection and would be expected to be temporary, abating when the infection or injury resolves. However a chronic state of inflammation can lead to an increased risk of cancer. This link is exemplified by the association of Helicobacter *pylori *(H. *pylori*) infection (causing chronic inflammation) and gastric cancer, the second most common malignancy worldwide [1,2]. Several other examples are documented, including chronic hepatitis B virus (HBV) infection and hepatocellular carcinoma [3] and the inflammation induced by chemical irritants (such as smoke or asbestos) with lung cancer.

Almost all cancers are accompanied by leukocyte infiltration, the significance of which has recently come under increasing scrutiny as to whether these cells work to eradicate the malignant cell, or whether they act to support it. Various inflammatory cell subsets are now thought to be able to contribute to tumour progression. The presence of innate immune cells such as granulocytes, dendritic cells, macrophages, natural killer cells and mast cells can functionally contribute to tumour development via the release of soluble factors which can mediate tumour-favourable processes including angiogenesis and tissue remodelling [4]. Additionally, soluble B-cell-derived factors have been shown to increase inflammatory cell recruitment and co-ordinately carcinogenic progression in a K14.HPV16:E6/E7 transgenic mouse model of epithelial carcinogenesis [5]. Furthermore, it is becoming increasingly clear that the ability of tumour cells themselves to secrete and/or respond to cytokines and chemokines can also provide a survival advantage [6].

Epstein-Barr virus (EBV) is associated with several malignancies, most tightly with the epithelial cancer nasopharyngeal carcinoma (NPC). NPC demonstrates an intense leukocyte infiltration within the tumour tissue, mainly composed of T-cells and macrophages and with the noted expression of interferon (IFN)-γ, BLC (CXCL13), CD40, interleukin-1 (IL-1), several macrophage inflammatory and chemoattractant proteins and in a small number of cases (10%) CD30 [7-10].

The EBV oncogene encoding latent membrane protein-1 (LMP1) has been shown to upregulate a number of cytokines and chemokines in various epithelial systems, including LMP1 transfected epithelial cell lines and gene expression correlated with LMP1 in NPC biopsies. These factors include IL-6, IL-1β, IL-1α, CXCR4, RANTES, MCP1, IL-8 and IL-10 [11-17]. Up-regulation of several factors by LMP1 has been shown to be mediated through its ability to activate NFκB signalling. NFκB has a dual role in carcinogeneis; its expression in potentially malignant cells can prevent cell death, additionally, it is a prominent mediator of inflammation, regulating the expression of pro-inflammatory cytokines such as IL-1, IL-6, IL-8 and tumour necrosis factor α (TNFα) [18,19].

In order to explore molecular and cellular processes in the very early stages of carcinogenesis, the link with chronic inflammation and the factors involved, we have used a transgenic mouse model of multistage epithelial carcinogenesis wherein LMP1 (of the nasopharyngeal carcinoma viral strain: LMP1^CAO^), is expressed in epithelia [20]. In this system we have previously shown that NFκB is activated by LMP1 *in vivo *[21].

In the present study we have gone on to characterise the inflammatory state in the effected transgenic skin and explored deregulated expression patterns, particularly those of cytokines and chemokines. The active role of adaptive immune cells in the inflammatory state in the model is demonstrated by the genetic removal of B- and T-cells using a RAG-1 null background, which limits the pathology to an early stage.

## Results

### Inflammation in the transgenic tissue

**Figure 1:**
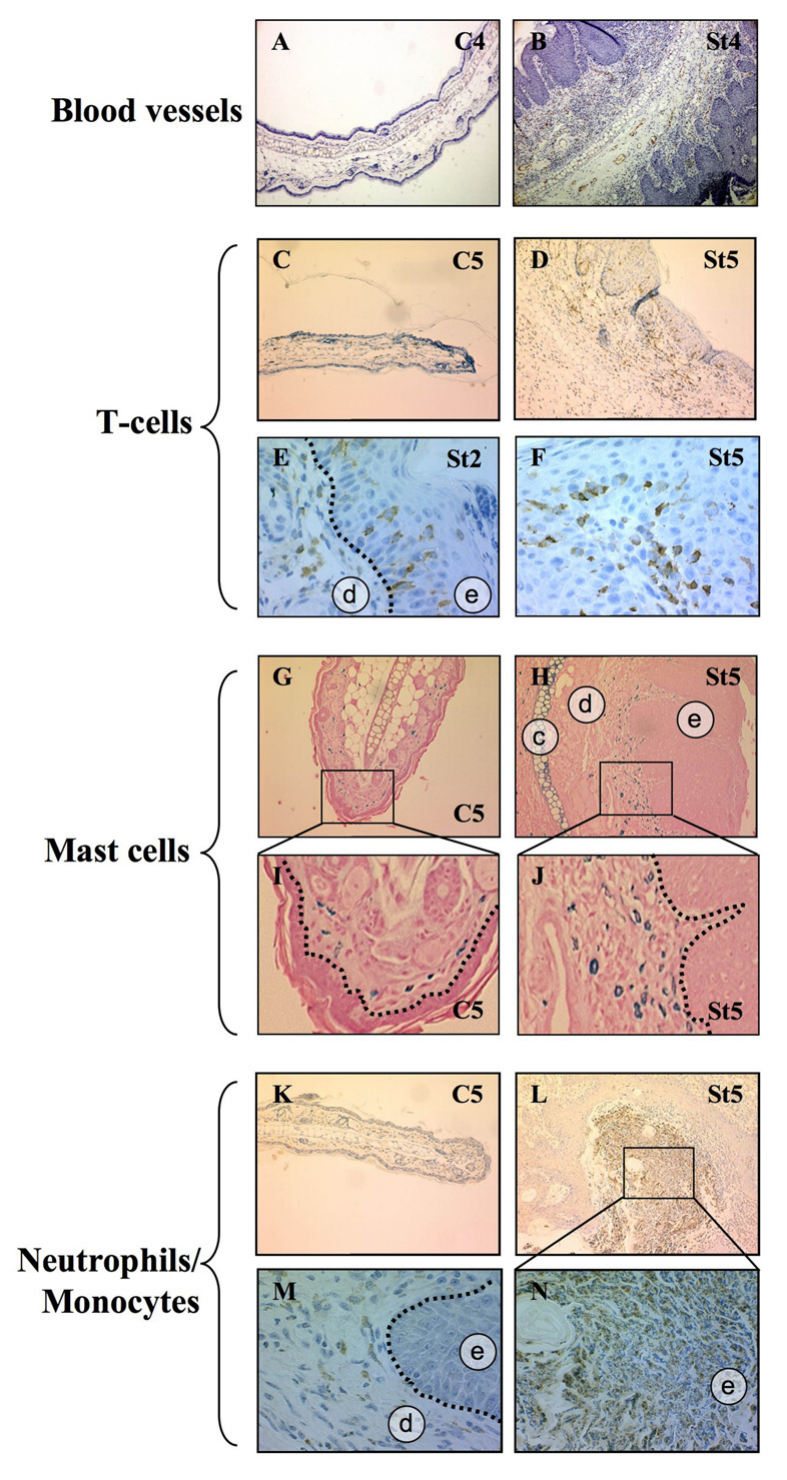
**Infiltrating cells in transgenic tissue**. Formalin fixed, tissue sections were immunostained with : **A-B**: anti-von Willebrand factor revealing blood vessels (brown stain) shown at original magnification x10 (see additional file 2, figure S1 for details); **C-F**: anti-CD3 to reveal T-cells (brown stain), at x100 (C, D) and x400 (E, F) original magnification; **G-J**: astra blue staining to reveal mast cells (blue) at x10 original magnification (G, H) with indicated areas enlarged in I and J; **K-N**: anti-myeloperoxidase to reveal neutrophils and monocytes (brown staining) at either x100 (K, L) or x400 (M) original magnification, with a region of the intensely stained epidermis (boxed in L) enlarged in (N). LMP1^CAO^.117 transgenic ear phenotypic stage 2 and 5 (St2 and St5 respectively) and NSC (C2 and C5) samples were used. The epidermis, **e**, dermis, **d**, and cartilage, **c**, are indicated. Dashed lines indicate the epidermal basement membrane.

L2LMP1^CAO ^mice have been previously described and show a hyperplastic phenotype in the skin, which progressively worsens as the mice age. The most striking phenotype presents in the hairless skin regions, particularly the ears of the mice (correlating with the levels of LMP1 transgene expression). This preneoplastic phenotype has been categorised into five recognisable and predictable stages, from stage 1 (St1) showing mild hyperplasia with increased vascularisation to stage 5 (St5) displaying severe hyperplasia with necrosis and tissue degeneration (figure 1A, B and detailed in additional file 1 table S1), which can lead to acanthosis, hyperkeratosis and occasional carcinoma [20]. First, the inflammation status was assessed in the tissue by examining infiltrating cell types by immunohistochemistry (IHC). Ear tissue from L2LMP1^CAO^.117 mice was analysed at stages 2 and 5, representing early and advanced pre-neoplastic pathology and compared to aged matched controls (C2 and C5 respectively). The tissues were examined for the presence of mast cells, neutrophils, T-cells, granulocytes and eosinophils. Differences were detected between transgenic and control tissues in the T-cell, mast cell and neutrophil/monocyte infiltrate (figure 1). T-cells were present in the dermis of both the transgenic and control tissue, however they were increased in number in the transgenic dermis and were also present in the transgenic epidermis (unlike the controls) at both early and advanced stages (figure 1, C-F). Increased numbers of mast cells were evident in the transgenic tissue compared to controls, localised in the dermis beneath the epidermal basement membrane whilst in the controls they showed a more scattered pattern (figure 1, G-J). Myeloperoxidase staining (used to detect neutrophils and monocytes) revealed some weak staining throughout the dermis of controls and transgenic samples, however, regions of intense staining in localised areas of the epidermis were detected in the transgenic tissue only (figure 1, K-N). In addition in the transgenic stage 4 and 5 tissue, swathes of degenerating neutrophils were apparent in areas of ulceration and necrosis. These findings are consistent with the pathological diagnosis indicating mixed inflammatory infiltrates including lymphocytes, neutrophils and mast cells with areas of degenerate neutrophils notably in tissue stages 3 to 5.

**Figure 2:**
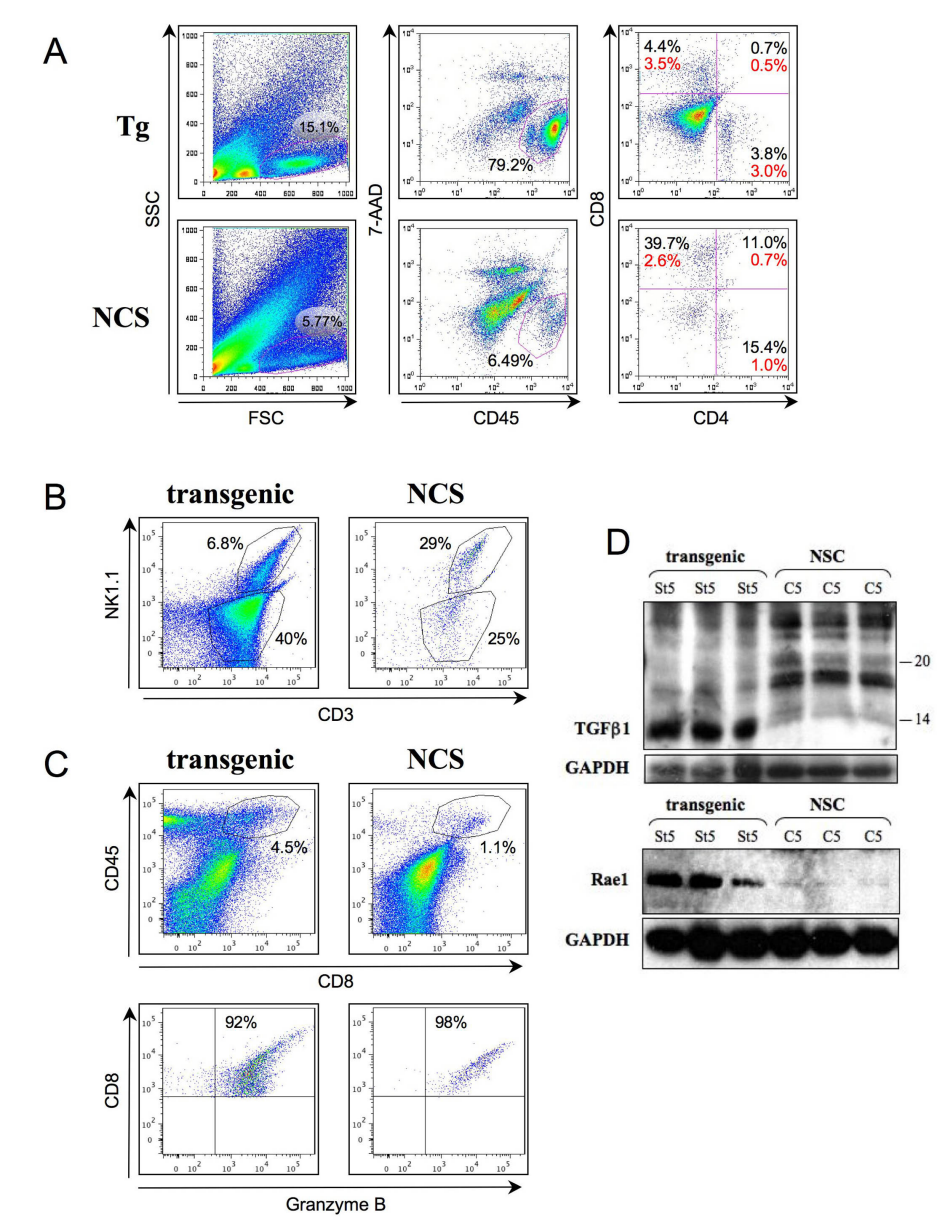
**Characterisation of the T-cell infiltrate**. **A, B, C**: Cell suspensions from transgenic St3/4 (Tg) and control (NSC) ear tissue (approximately 10^6 ^cells/sample, each from two transgenic ears or 4 to 6 control ears) were analysed by flow cytometry using fluorescent-conjugated antibodies. In all cases, the leukocyte populations were first gated by forward and side scatter as shown in the first panel of **A**. Viable leukocytes were further gated as the CD45^+^(APC)/7-AAD^-^(PerCP) population shown in the middle panel of **A**. Cells were further stained using antibodies directed to CD4(PE) and CD8(FITC) (**A**), NK1.1(PE) and CD3(FITC) (**B**) and CD8(FITC) and Granzyme B(PE) (**C**); showing proportions of cells in relevant quadrants or gates. CD4/CD8 proportions in (**A**) are given as the % of CD45^+^/7-AAD^- ^cells (black) and as the % of the gated FSC/SSC cells (red). **D**: Protein was extracted from control (NSC: C5) and transgenic ears (St5). Three biological replicates (40 μg) were western blotted and probed with anti-TGFβ1 (upper panel: 15% gel, precurser dimer: 44kD, mature protein dimer: 25kD, under these reducing conditions: 22 and 12.5kD respectively) or α-Rae1 (lower panel: 12.5% gel, migrates at ~40kD). α-GAPDH was used as a loading control as indicated (37kD).

To characterise the leukocyte subsets within the ear tissue, a cell isolation protocol was used to disassociate the cells for flow cytometry, avoiding the use of trypsin and prolonged dispase treatment which can impair surface marker detection [22]. In reflection of the hyperplastic pathology, two to three times as many non-transgenic sibling control (NSC) ears compared to transgenic samples were required to obtain sufficient cell numbers for this purpose. In agreement with the IHC analysis, a greater proportion of CD45^+ ^leukocytes were present in the transgenic ear tissue compared to the controls (representative example in figure 2A) with between 60% and 80% CD45^+ ^cells (of the FSC/SCC gated population) in the transgenic samples compared with 2% to 7% in NSC samples (reverse gating showed that all viable CD45^+ ^cells reside within this FSC and SCC gated population). Of the CD45^+ ^gated populations 47% were CD3^+ ^T cells in the transgenic samples and 54% in the control samples (bearing in mind the approximately 30 fold smaller CD45^+ ^population in the controls) (figure 2B). In the transgenic samples, 6.8% (of CD45^+ ^cells) were CD3^+^NK1.1^+ ^(indicative of a subset of NKT cells), the vast majority of the T-cells (CD3^+^) being NK1.1^- ^(figure 2B). In the controls 29% (just over half of the T-cells present) were CD3^+^NK1.1^+^. Despite the greater ratio of CD3^+^NK1.1^+ ^to CD3^+^NK1.1^- ^cells in the control tissue compared to the transgenic, this represents approximately 8 fold fewer NKT cells per control ear compared to the transgenic ear. NKT cells can secrete transforming growth factor β (TGFβ), which is a positive signal for their proliferation yet an inhibitory factor for their cytotoxic activity. In accordance with this, elevated levels of mature (processed) TGFβ1 (figure 2D), but not β2 or β3 (not shown) were observed in the transgenic St5 samples. No NK1.1^+^CD3^- ^cell population (indicative of NK cells) was apparent in either transgenic or NSC samples (figure 2B). Moreover, elevated Rae-1 levels were observed in St5 samples compared to controls (figure 2D). Rae-1 is a ligand which activates NK cells (NK1.1^+^CD3^-^) via the NKG2D receptor, however sustained elevation of Rae1 results in impaired NK cell function and a subsequent decrease in anti-tumour immunosurveillance [23]. Thus we would predict that the inflamed transgenic tissue environment would be inhibitory for NKT and NK cytotoxic activities.

**Figure 3:**
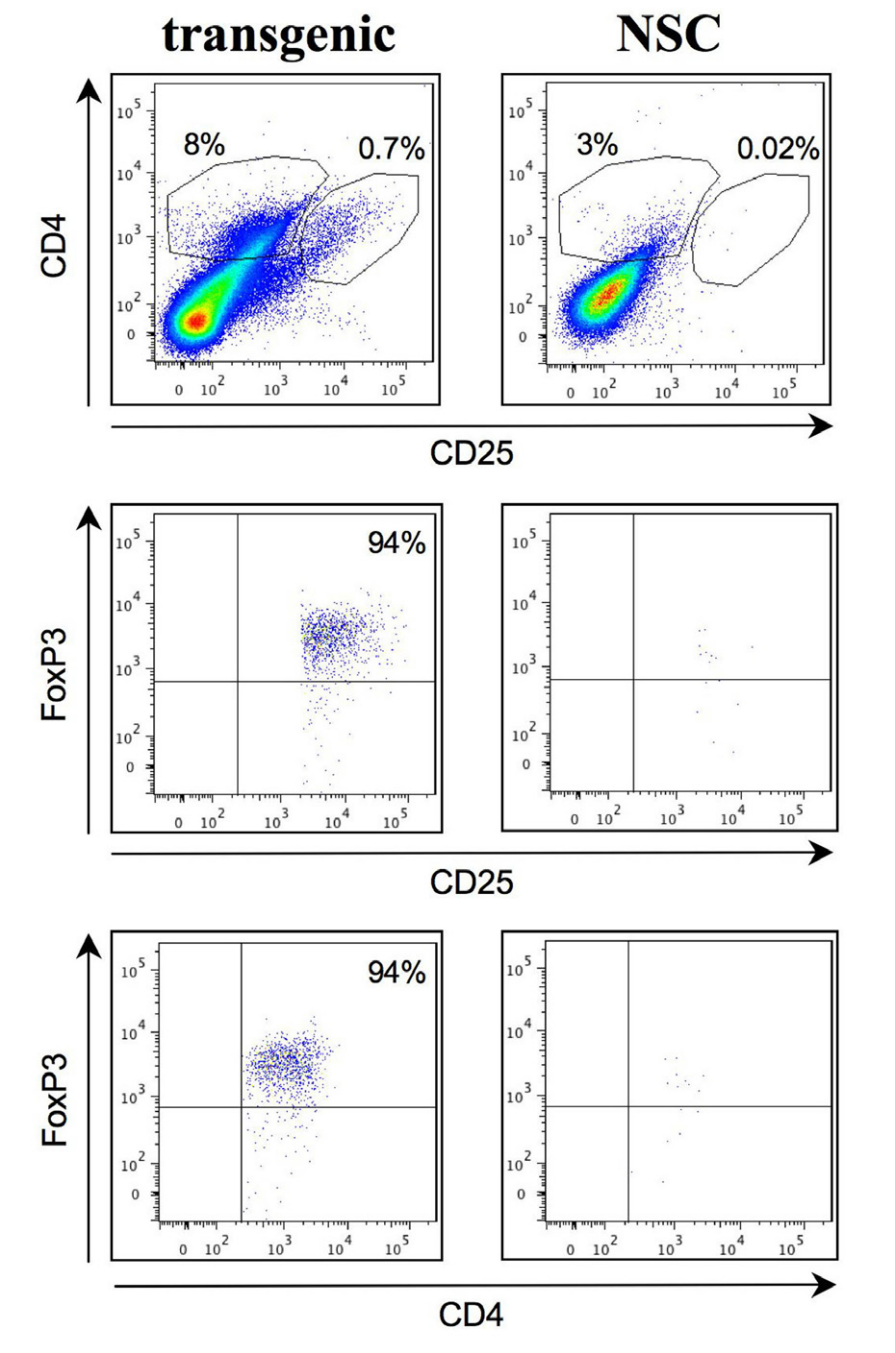
**Increased Treg cells in the transgenic tissue**. Cell suspensions from transgenic St3/4 and control (NSC) ear tissue (approximately 5 × 10^5 ^cells/sample) were analysed by flow cytometry using fluorescent-conjugated antibodies. The leukocyte populations were first gated by forward and side scatter as shown in figure 2. Cells were stained using antibodies directed to CD4(FITC), CD25(PE) and FoxP3(APC). CD4^+^CD25^+ ^cells gated in the upper panel are shown for FoxP3 staining in the middle and lower panels. The proportions of cells in relevant quadrants or gates are given.

Further characterisation of the T-cell subsets revealed that 7% were positive for CD4 and/or CD8 (of the FSC/SSC gated population) in the transgenic samples compared to 4.3% in controls (figure 2A). In addition, the transgenic samples showed a large proportion of leukocytes negative for CD4 and CD8, presumably including the mast cells and neutrophils noted above. The CD8:CD4 ratio for transgenic compared to NSC was 1.2 and 2.6 (respectively), suggesting a relative increase in the CD4^+ ^population in the transgenic samples (figure 2A). Co-staining of the CD8^+ ^populations revealed that virtually all were granzyme-B^+ ^(figure 2C) in both transgenic and control samples, indicating that the cytotoxic T-cells within the ear tissue are activated and that this is normal, although there are more in the transgenic tissue compared to the controls. No CD8^+ ^cells were found to co-stain with CD25 and FoxP3 (not shown). Analysis of the CD4^+ ^cells revealed a proportion (approximately 9% of the total CD4^+ ^population) in the transgenic samples co-staining for both CD25 and FoxP3, indicative of Treg cells, while no such population was apparent in controls (figure 3). Thus the transgenic samples show increased numbers of CD4^+ ^T-cells with an increased proportion of Treg cells compared to controls.

### Immunoglobulin deposition in the transgenic tissue

**Figure 4:**
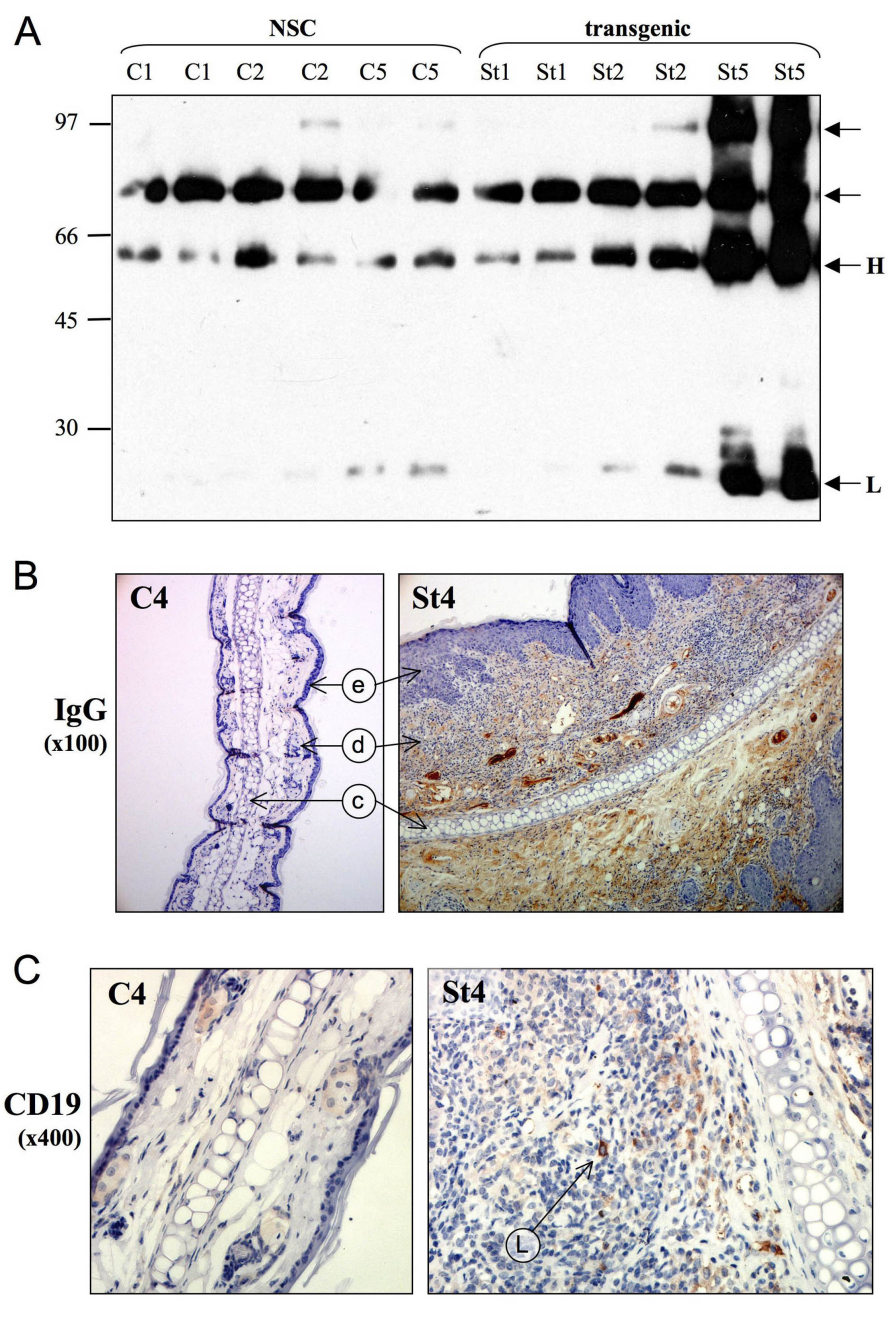
**Immunoglobulin deposition in the transgenic dermis**. **A**: Protein was extracted from control (NSC: C1, C2, C5) and transgenic ears (St1, St2, St5). Two biological replicates (40 μg) were separated by 10% SDS-PAGE, western blotted and probed with goat α-mouse IgG-HRP. The immunoglobulin heavy (H) and light (L) chain bands are indicated at 55kD and 25kD respectively. Molecular weight markers are shown (kD). **B **and **C**: Ear tissue sections for transgenic St4 and NSC (C4) were immunostained with goat α-mouse IgG shown at original magnification of x100 (B) and anti-CD19 detecting B-cells at x400 (C).

Immunoglobulin deposition is a recognised feature of several chronic inflammatory disorders, such as rheumatoid arthritis and the active role of B-cells in autoimmune disease is evidenced by a decrease in disease severity following B-cell depletion in patients [24]. Immunoglobulin deposition and a B-cell role in disease is also proposed for several carcinomas, including breast and prostate cancer and was observed experimentally in the skin of human papillomavirus 16 (HPV16) transgenic mice [25]. In order to determine if immunoglobulin deposition also occurs in the L2LMP1^CAO ^mice, the ear tissues were examined by western blotting and IHC. Biological replicates of transgenic tissue at stages 1, 2 and 5 along with controls revealed heavy and light chain IgG antibody bands in all samples, these were slightly increased in St2 samples compared to controls but were markedly more intense in the St5 extracts (figure 4A). By IHC, the IgG deposition was observed to be pronounced throughout the dermis of the transgenic tissue and not in controls (figure 4B and additional file 2 figure S2). In order to assess if B-cells were infiltrating the tissue (although this is not necessary for immunoglobulin deposition to occur), sections were immunostained with antibodies to CD20 and CD19. No staining was observed in the skin samples using anti-CD20, however, very sparse positively stained lymphocytes with plasma cell appearance in the transgenic dermis were apparent using anti-CD19, while no specific staining could be detected in controls (figure 4C).

### The inflammatory environment in the transgenic tissue

The transgenic tissue clearly shows considerable inflammatory cell infiltration. In order to gain a broad overview of the status of inflammatory factors in the transgenic tissue environment, cytokine and chemokine levels were examined in both serum and ear tissue of L2LMP1^CAO^.117 and NSC mice using a multiplexed immunodetection array (table 1, the full list tested is shown in additional file 1 table S2). Serum and ear tissue from St5 phenotype mice and ear tissue from St2 phenotype mice were compared with C5 and C2 (respectively), pooling four samples in each group. Of the cytokines known to be influenced by LMP1 expression in other systems, IL-4 and IL-6 showed no difference between transgenic and NSC in either serum levels or in the pathological tissue extract (additional file 1 table S2). Similarly, TNFα was not obviously induced in the transgenic samples, however one of its receptors, TNFRII, was detected at higher levels in the St2 tissue sample. The multifunctional factor IL-10 (induced by LMP1 in B-cells [26,27]), was detected at approximately 2 fold lower levels in the serum (compared to NSC), but approximately 2 fold higher levels within the affected tissue. The chemokine IL-8, through binding to the receptors CXCR1 and CXCR2 recruits and activates neutrophils, and its induction is associated with LMP1 in NPC [13,28]. Rodents lack a direct homologue of IL-8, however the chemokines CXCL1/KC, CXCL2/MIP2 and CXCL5-6/LIX are regarded as functional analogues [29]. Like IL-10, KC was detected at approximately 2 fold lower levels in the serum (compared to NSC), but approximately 2 fold higher levels within St2 tissue. MIP-2 was observed at 4.2 and 2.8 fold higher levels (St2 and St5 respectively) in the transgenic tissues and LIX at 3.7 and 2.2 fold higher levels (respectively), again without increase in the serum. Thus all three IL-8 murine analogues were observed at higher levels in the LMP1 affected transgenic tissue. IL-1β was found at 2 to 3 fold higher levels in the transgenic samples, but not IL-1α, which was at lower levels in the transgenic tissue.

**Table 1:** Cytokines and chemokines showing greater than 2 fold difference between transgenic and NSC samples

Serum St5/C5		Tissue St2/C2		Tissue St5/C5	
**cytokine**	**ratio**	**cytokine**	**ratio**	**cytokine**	**ratio**
					
CD30	16.49	CD30L	∞	CD30L	∞
TIMP-1	9.01	CD30	∞	CD30	∞
MIP-1γ	6.06	CXCL13(BLC)	18.06	CXCL13(BLC)	18.42
CD30L	4.17	CXCL10(CRG-2)	11.86	CXCL10(CRG-2)	11.47
Leptin	3.20	CD40	8.80	MIP-3α	7.09
IL-3Rβ	2.62	L-Selectin	8.50	CD40	5.21
Eotaxin	2.48	IL-3	6.11	IL-12P40/P70	5.17
FasLigand	2.27	MIP-2	4.20	IL-3	4.60
XCXL10(CRG-2)	2.19	MIP-3β	3.92	L-Selectin	4.19
RANTES	2.06	sTNF RII	3.83	MIP-3β	3.23
CTACK	2.05	IL-12P40/P70	3.76	IL-2	3.10
				
		IL-3 Rβ	3.73	MIP-2	2.84
				
TARC	0.50	LIX	3.72	CXCL16	2.70
IGFBP-3	0.48	IL-2	3.39	AXL	2.61
MIP-3β	0.46	MIP-3α	3.30	IL-3 Rβ	2.30
KC	0.46	TIMP-1	3.14	GCSF	2.28
IL-12p40/p70	0.46	IL-1 β	3.09	VEGF	2.25
IL-13	0.43	IGFBP-5	2.95	LIX	2.23
MIP-3α	0.35	TPO	2.85	IFNγ	2.19
IL-17	0.25	IL-13	2.71	GM-CSF	2.18
IFNγ	0.23	IL-17	2.69	IL-1 β	2.16
				
IGFBP-5	0.20	GCSF	2.42		
				
TARC	0.50	VEGF	2.41	IL-1α	0.38
IGFBP-3	0.48	VCAM-1	2.30	IGFBP-6	0.36
MIP-3β	0.46	PF-4	2.19		
KC	0.46	KC	2.11		
IL-12p40/p70	0.46	AXL	2.10		
IL-13	0.43	IL-10	2.10		
		Leptin R	2.05		
		GM-CSF	2.04		
		IFNγ	2.00		
				
					
				
		IGFBP-6	0.37		
		eotaxin2	0.31		

A pool of four samples (from different mice) were used for each sample assayed by cytokine immunodetection array. Samples were taken from serum from transgenic mice with St5 ear phenotype and serum from non-transgenic sibling controls (NSC) (1^st ^column), transgenic stage 2 ear tissue extracts (St2) compared with C2 (2^nd ^column) and transgenic stage 5 ear tissue extracts (St5) compared with C5 (3^rd ^column). The mean of the normalised values obtained for each cytokine was taken and the ratio of transgenic over NSC determined. All those factors showing greater than two fold difference are shown. For CD30 and CD30L (CD153), strong signal was detected in the transgenic and none in the control samples, as such the ratio cannot be determined (∞).

**Figure 5:**
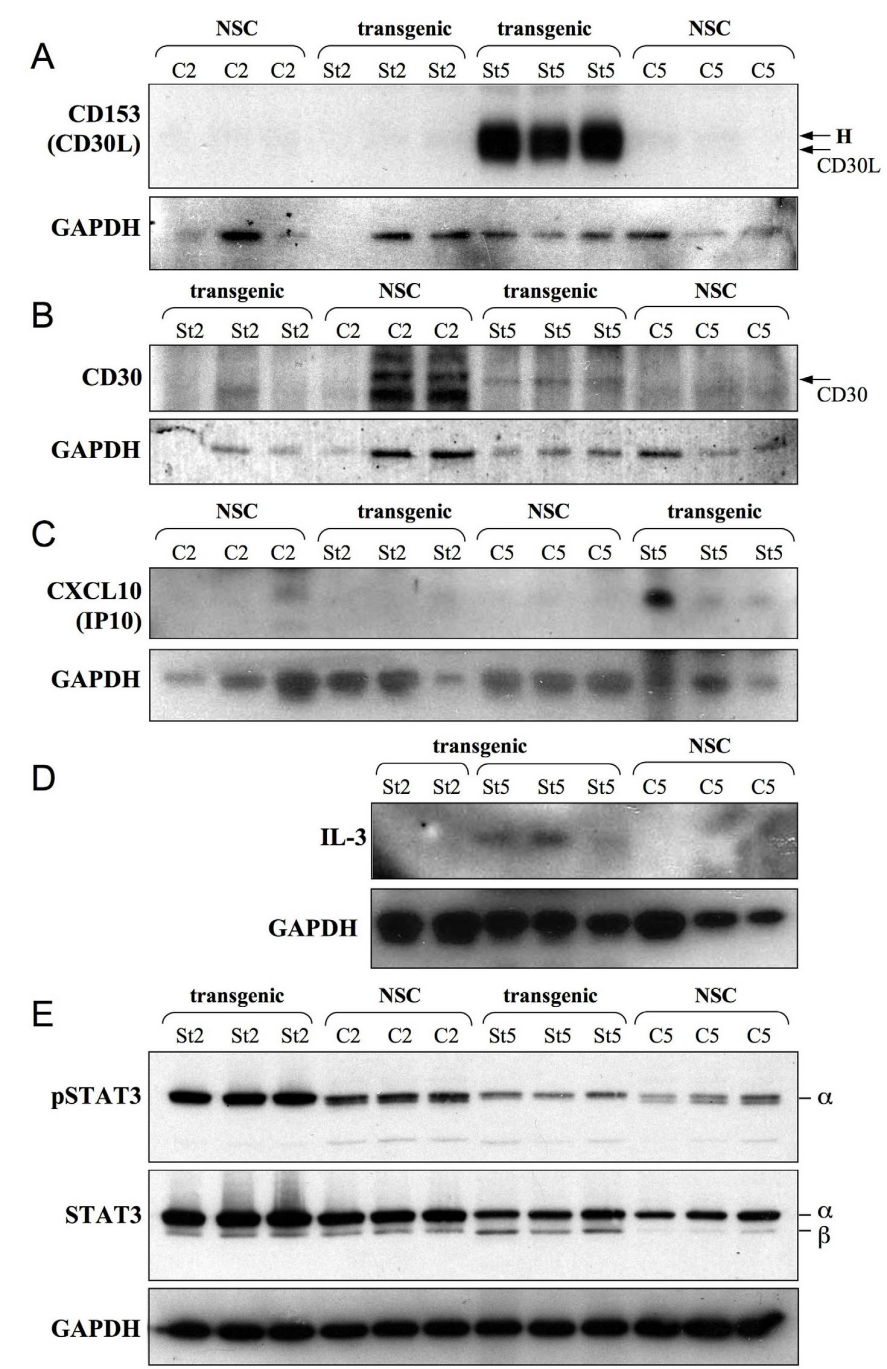
**STAT3 is activated in the transgenic tissues and inflammatory factors induced**. Protein was extracted from control (NSC: C2, C5) and transgenic ears (St2, St5). Three biological replicates (100 μg) were separated by SDS-PAGE and western blotted. **A**: 10% gel probed with α-CD153 (migrates at ~44kD); **B**: 10% gel probed with anti-CD30 (migrates at 120kD), note the non-specific bands in two C2 samples due to relatively high loading, with no CD30 specific band; **C**: 15% gel probed with anti-CXCL10 (migrates at 10kD); **D**: 15% gel probed with anti-IL-3 (migrates at 15kD); **E**: 10% gel probed sequentially with anti-phospho-ser727-STAT3 (migrates at 89kD) then anti-total-STAT3 (α isoform migrates at 86kD, β isoform migrates at 79kD). Detection of GAPDH was used as a loading control (37kD) shown below each panel.

**Figure 6:**
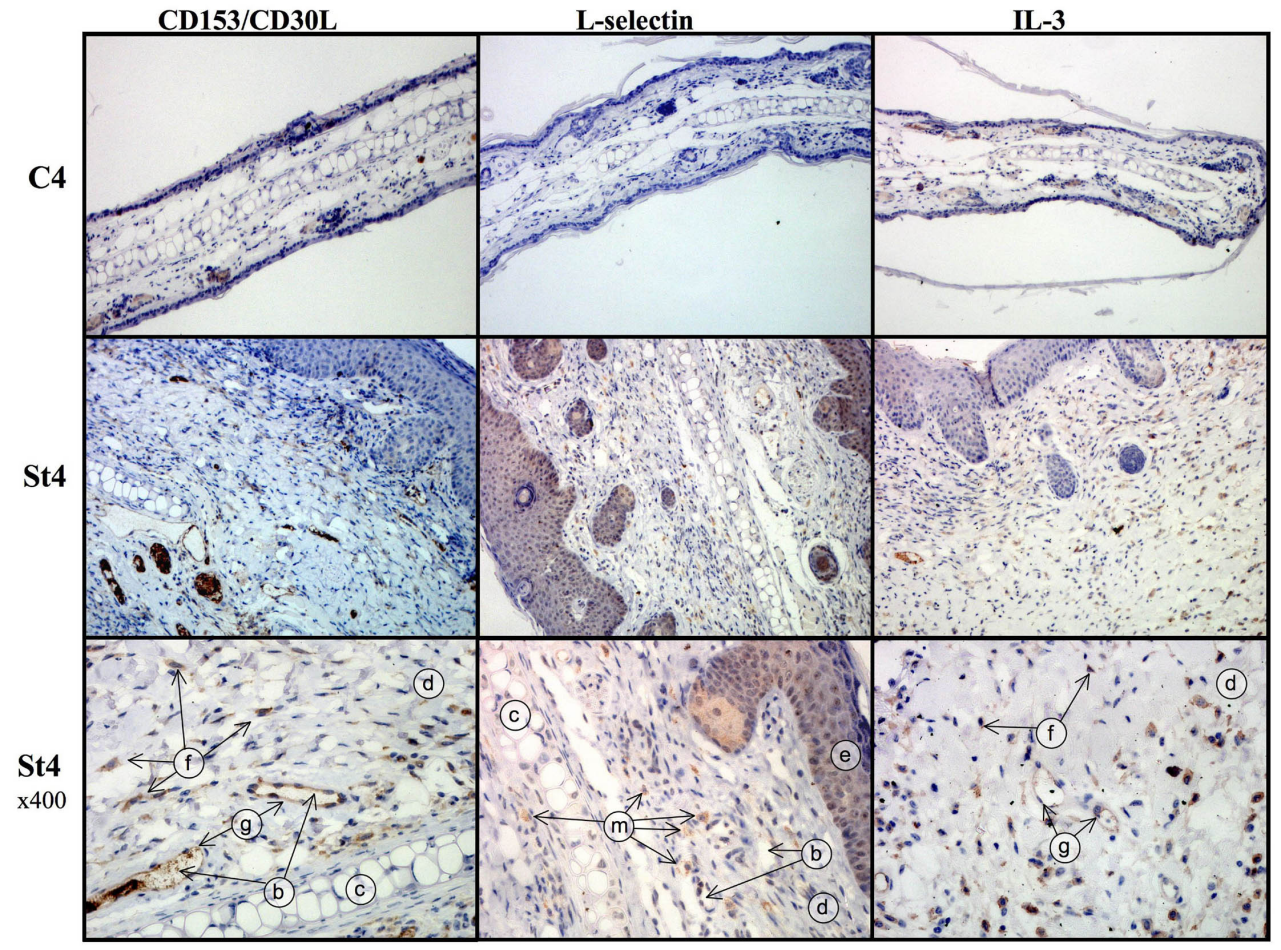
**Expression of CD153, L-selectin and IL-3 within the transgenic tissue**. Representative formalin fixed, tissue sections from LMP1^CAO^.117 transgenic ear phenotypic St4 and NSC (C4) were immunostained with anti-CD153, anti-L-selectin and anti-IL-3 (brown stain) as indicated. Original magnification x200 is shown in the upper two panels with x400 of the St4 sections in the lower panel (further micrographs are shown in the supplementary information). Some example features are identified: b = blood vessels, c = cartilage, d = dermis, e = epidermis, f = positive staining in fibroblasts, g = positive staining in vascular endothelial cells, m = granular positive staining in the cytoplasm of mast cells.

Of the factors analysed in the array, those showing the greatest upregulation in the transgenic samples compared to NSC in tissue extracts (and in some cases serum also) were CD30 and its ligand CD153 (CD30L), CXCL13 (BLC), CXCL10 (CRG-2 or IP10), CD40, L-selectin and IL-3. Expression in the tissues of these factors was explored further by western blotting and IHC. In some cases the data were ambiguous due to cross-reactivity detected by the available antisera. However, clear upregulation in the transgenic St4 and St5 tissue of CD153 (CD30L), a costimulatory molecule expressed by activated B- and T-cells, mast cells and macrophages, was detected (figure 5). No CD153 was detected by western blotting in the control tissues (figure 5) and very little immunohistochemical staining was observed (figure 6 and additional file 2 figure S3). In the transgenic tissue CD153 was observed in the cytoplasm of infiltrating inflammatory cells, probably mast cells and fibroblasts as well as intense staining in vascular endothelial cells, which was not detected in NSC tissue sections (figure 6). CD30 was also confirmed as upregulated by western blotting in St5 extracts (figure 5), but due to antibody cross reactivity, specific staining could not be determined in tissue sections. L-selectin is an adhesion molecule which is usually expressed on the surface of leukocytes and mediates their migration from the blood stream. By IHC, L-selectin was observed in the transgenic tissue, with weak staining in nuclei and cytoplasm of the epidermal cells (figure 6 and additional file 2 figure S4). Some weak staining in the nuclei of control epidermal cells was also seen, which may reflect non-specific staining. Specific staining for L-selectin was observed in the transgenic tissues within mast cells in a clear granular pattern indicative of L-selectin present within the mast cell granules (figure 6 and additional file 2 figure S4). Rare cells stained for L-selectin in the NSC tissues. IL-3, a potent growth promoting cytokine, was observed to be upregulated at St5 but not St2 by western blotting with none detected in controls (figure 5). IL-3 immunostaining was detected in the transgenic tissue in fibroblasts, infiltrating cells and in vascular endothelial cells, but not in controls (figure 6 and additional file 2 figure S5). CXCL10 (or CRG-2 or IP10) is an IFNγ responsive chemokine with pleiotropic affects. Binding to its receptor (CXCR3) can induce T-cell migration, modulation of adhesion molecule expression and monocyte and NK cell stimulation. CXCL10 showed an 11 fold increase in the transgenic tissue compared to controls by the array and was confirmed to be upregulated in the transgenic St5 tissue by western analysis (figure 5).

Several members of the macrophage inflammatory protein group showed considerable upregulation in the transgenic samples by the array analysis, specifically macrophage inflammatory protein-1γ (MIP-1γ) in the serum (which has been confirmed by ELISA - not shown), MIP2 (CXCL2, discussed above), MIP-3α and MIP-3β in the tissues. Additionally IFNγ, found induced in NPC tissues, was detected at approximately 2 to 3 fold higher levels in the St2 and St5 tissues, with reduced levels in serum compared to controls, a pattern also observed with IL-10 and the murine IL-8 analogues. The cytokines IL-12 (proposed to be induced by LMP1 in B-cells [30]), IL-2, IL-3 (and receptor IL-3Rβ) and the pro-inflammatory IL-1β were detected at higher levels in St2 and St5 tissues than controls. The angiogenic factor vascular endothelial growth factor (VEGF) was also detected at higher levels in the tissue samples (2x) and was previously observed to be induced in the transgenic samples by western blotting [20]. Members of the insulin-like growth factor binding protein group (IGFBP) were amongst the few factors showing reduced levels in the transgenic serum and tissues by the array analysis (table 1 and additional file 1 table S2).

It is becoming increasingly apparent that signal transducer and activator of transcription-3 (STAT3) is a seminal factor in inflammatory processes. Persistent activation of STAT3 has been linked with tumour-associated inflammation and suppression of anti-tumour immunity [31]. STAT3 has two isoforms (α and β) which show differences in function [32]. STAT3 expression and activation (by phosphorylation at serine 727, which affects DNA binding and transcriptional activity) were examined in the transgenic tissues compared to controls (figure 5D). STAT3α was the predominant form expressed in transgenic and control ear tissues. A lower level of STAT3β was detected in the transgenic and control young mice (St2 and control), however in the older mice (St5 at 6 months), the β form was reduced in controls, but not in transgenic samples. Increased levels of activated (ser-727-phosporylated) STAT3α was detected in the transgenic St2 samples compared to controls, but at the later St5 there were equivalent levels to controls (figure 5D). Interestingly, a doublet of phosphorylated STAT3 was observed in all control samples, each band of the doublet at roughly equal intensity, while only the upper band (STAT3α) was observed in the transgenic samples. The lower phosphorylated band of the doublet, not observed in the transgenic samples, is presumably the phosphorylated STAT3β isoform. Thus STAT3 is activated in the transgenic samples compared to controls at an early stage (St2) during the onset of the inflammatory pathology and the two isoforms are differentially regulated at the later stages.

### The contribution of B- and T-cells to the phenotype

**Figure 7:**
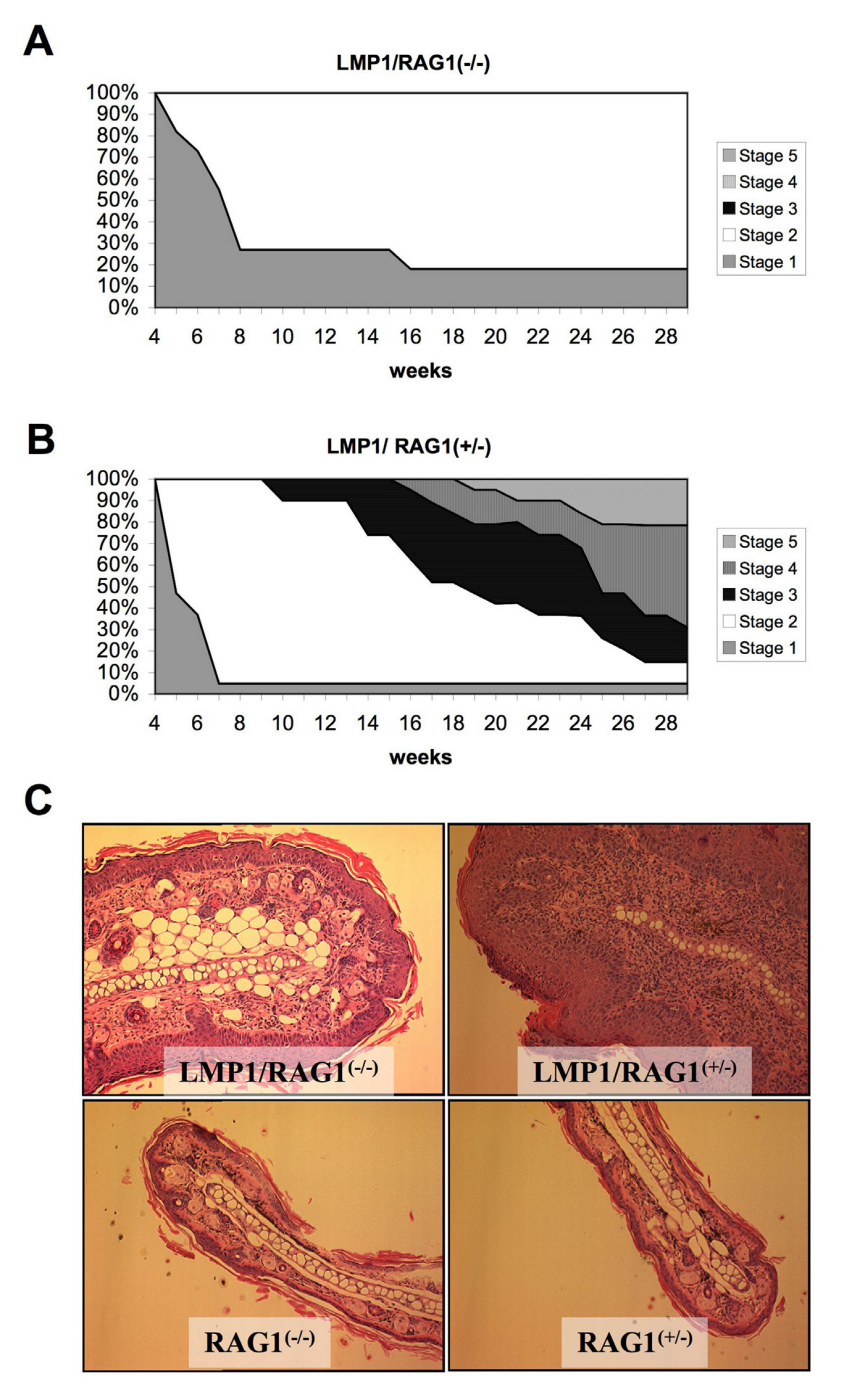
**Phenotype limitation in a B/T-cell deficient background**. **A **and **B**: Two cohorts of LMP1 transgenic mice, in either a RAG1 null (-/-), n = 11 (**A**) or RAG1 heterozygote, n = 18 (+/-) (**B**) background were monitored weekly (from 4-29 weeks old). The phenotypic stage of the ears of each mouse was recorded. Two dimensional area plots represent the percentage of mice at each ear stage (*y *axis) at weekly intervals (*x *axis). **C**: H&E staining of tissue sections from the ears of RAG1 null or RAG1 heterozygote mice in the presence or absence of the L2LMP1^CAO ^transgene, as indicated. Original magnification: x100. Note, mice in this study were all 75% C57Bl/6/25% FVB strain.

**Figure 8:**
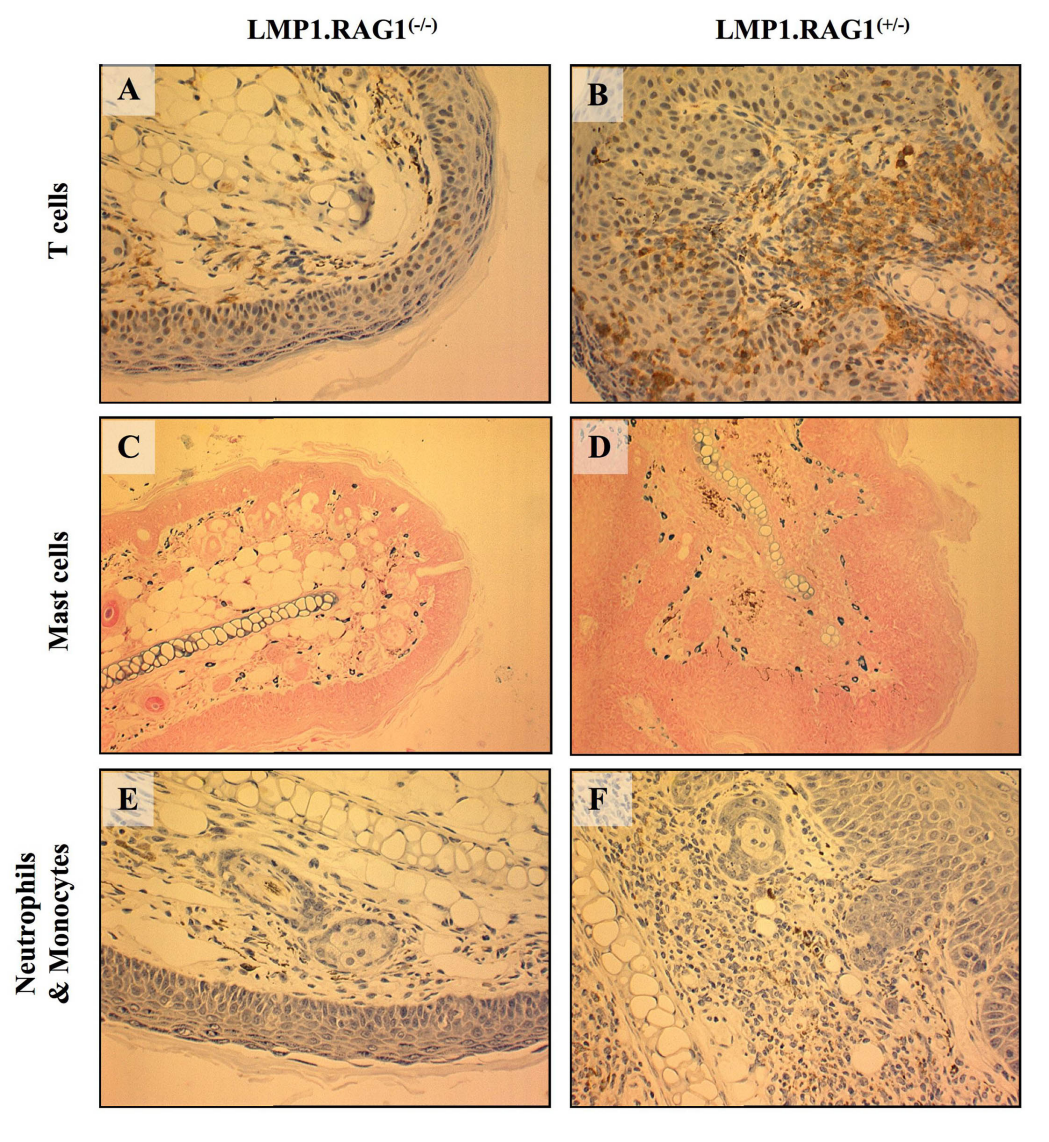
**Reduced inflammation in a RAG1 null background**. Immunohistochemical analyses of ear tissue sections from LMP1^CAO^.117 transgenic mice in a RAG1-null background (LMP1.RAG1^(-/-)^) compared to RAG1 heterozyous background (LMP1.RAG1^(+/-) ^Formalin fixed, tissue sections were immunostained with: **A, B**: anti-CD3 to reveal T-cells (brown stain), at original magnification: x200; **C, D**: astra blue staining to reveal mast cells (blue) at original magnification: x100 **E, F**: anti-myeloperoxidase to reveal neutrophils and monocytes (brown stain) at original magnification: x100.

We next explored if adaptive immune cells present in the phenotypic tissue contribute to the LMP1 induced pathology. L2LMP1^CAO^.117 mice were bred into a RAG1-null background (lacking mature B-cells, T-cells and NKT-cells). LMP1/RAG1-null (n = 11) were compared to LMP1/RAG1-het (n = 18) over a 6 month period from birth (figure 7). The ear phenotype was staged 1-5 on a weekly basis. Within the time scale of the study, the majority of LMP1/RAG1-het mice reached at least St3 phenotype (17/18) and most reached St4 (14/18) with a proportion reaching St5 (5/18) (figure 7), following a phenotypic progression indistinguishable from the LMP1 mice in a wild type background (not shown). In contrast, none of the LMP1/RAG1-null mice passed St2 of the phenotype (epithelial hyperplasia) with 2/11 animals failing to advance beyond St1. The difference over time to develop each stage of the phenotype was highly significant between the two populations (St2: p = 0.009; St3: p < 0.0001, St4: p = 0.0033). Histopathology of tissues at the end of the study period (6 months) confirmed the staged observations, revealing a mild hyperplasia in the LMP1/RAG1-null St2 tissues compared to the typical St4 pathology in the LMP1/RAG1-het St4 tissue (figure 7C). Analysis of T-cell infiltrate shows the presence of T-cells in the LMP1/RAG1-het tissue and confirms the absence of T-cells in the LMP1/RAG1-null tissue (figure 8A, B). Similarly, the degree of mast cell infiltration in the LMP1/RAG1-null tissue is less than that observed in the LMP1/RAG1-het littermates (the former is similar to LMP1-St2 ears in a wild type background), whilst the LMP1/RAG1-het tissue displays mast cells localised beneath the dermal/epidermal basement membrane as observed for St4 and St5 L2LMP1.117 in a wild type background (Figure 8C, D). Lastly, the number of dermal neutrophils/monocytes is also fewer in the LMP1/RAG1-null compared to the LMP1/RAG1-het tissue. Thus, the presence of B- and/or T-cells is required for the phenotype to advance from the initial state of hyperplasia to severe, inflamed hyperplasia with necrosis and tissue degeneration from which keratoacanthoma and other neoplasms arise.

## Discussion

Extensive leukocyte infiltration is a feature of several cancers, including the EBV associated malignancies NPC, Hodgkin's Disease (HD) and gastric cancer. We have used a model of epithelial carcinogeneisis, transgenic mice expressing the primary oncogene of EBV, LMP1, to explore the inflammatory processes prior to neoplasia. The ears of the L2LMP1^CAO ^mice (the tissue where LMP1 is expressed at the highest level) and to a lesser extent other regions of body skin develop a preneoplastic pathology of hyperplasia with increased vascularisation, progressing to acanthosis, hyperkeratosis, parakeratosis and erosive or ulcerative dermatitis, which can lead to the development of keratoacanthoma, papilloma and ultimately carcinoma. Examination of the preneoplastic stages has revealed that the tissue is inflamed, with infiltrates of T-cells, mast cells and neutrophils, that occasional plasma cells are observed and IgG is deposited in the dermis and that several cytokines and chemokines involved in inflammation are induced. The increased numbers of T-cells in the transgenic tissue include both CD8^+ ^and CD4^+ ^cells, with a bias towards the latter as well as the induction of CD4^+^/CD25^+^/FoxP3^+ ^Treg cells.

We have previously reported the deregulation of proteins involved in hyperproliferation, inflammation, metastasis, angiogenesis and oxidative stress in the LMP1 expressing transgenic tissue [20,21,33] and now show the induction of further inflammatory chemokines and cytokines. The consequence of this LMP1-initiated expression programme *in vivo *is a hyperplastic tissue which is chronically inflamed and is predisposed to carcinogenesis. Several genes found to be up or down-regulated in LMP1 expressing tumour tissues and in the L2LMP1^CAO ^transgenic model, will result from a cascade of events due to the multiple cell interactions within a complex tissue, initiated by LMP1 but not necessarily direct targets of LMP1 signalling. In addition, gene expression changes within the tissue could originate from either the neoplastic cell, the leukocyte infiltrate or the stroma and thus would not necessarily be detected in a cultured clonal cell line. Nevertheless, expression of LMP1 was found to induce sets of genes involved in proliferation and inflammation in the SCC12F carcinoma cell line [16]. Upregulation of IL-1β and CD40 have been found in common in the SCC12F cell line system [16], in NPC tissues [7] and in our transgenic model. Additional proteins found to be upregulated in EBV associated disease, such as CXCL13 in NPC and CD30 in Hodgkin's disease (HD) (and in some NPC), were also detected at high levels in the transgenic tissue, but not in the SCC12F cell line system, suggesting that these may result from *in vivo *interactions. Therefore transgenic mouse epithelial expression of LMP1 represents a valuable model to study the LMP1 induced deregulated cellular expression programme and the consequences this has upon the cell, its environment and the tissue as a whole.

A key advantage of this *in vivo *model is that we can examine the sequential changes through time as the pathology develops from pre-neoplastic stages through to malignancy. Expression changes noted in the very early stages in young mice (St1 and St2) may result directly from LMP1 activation of target signalling pathways and be causal in the phenoytpe. Conversely, expression differences noted in the later stages only (St4, St5, papilloma and carcinoma), are likely to result from the consequences of the earlier altered expression programmes, such as the infiltration of inflammatory cells, and act to compound the phenotype. We previously found that TGFα and other epidermal growth factor (EGFR) ligands (including HB-EGF and EPR) were consistently upregulated in the transgenic tissue from the earliest stages, indicating that induction of these ligands result directly from LMP1 expression [20,21], furthermore increased serum TGFα has been correlated with poor prognosis in NPC patients [34]. EGFR is a known target of LMP1 through NF-κB activation [35,36] and we found that EGFR was induced (and NF-κB activated) by LMP1 in the transgenic tissue, but subject to homeostatic modulation *in vivo*, mediated in part through TGFα [21]. STAT3, like NF-κB, is a key regulator of inflammatory processes and frequently activated in cancer [31]. It has been proposed that LMP1 induction of EGFR is mediated by STAT3 through Bcl-3 in conjunction with NF-κB [37]. In the reciprocal process, signalling through EGFR can activate STAT3 [38], producing a feed forward loop between these factors. Moreover, nuclear EGFR and STAT3 physically interact to activate gene expression [39]. Increased STAT3 activation by phosphorylation was detected in the tissues of young transgenic mice (St2) compared to controls, suggesting an early role in the phenotype. By the later St5, phosphorylated STAT3α levels were similar to controls, at a point when EGFR levels are greatly reduced in the transgenic tissue [21], potentially reflecting a synergy between these factors *in vivo*. However in the St5 samples there are clearly differences in the regulation of STAT3 compared to controls. Higher levels of STAT3β are evident in the transgenic St5 samples, while in the controls the anti-phospho-ser727 antibody reveals a STAT3 doublet not apparent in the transgenic tissues. The STAT3β isoform results from a splice variation and lacks the C-terminal transactivation domain present in STAT3α and has been found to have different nuclear retention properties from STAT3α and different function [32,40]. The relevance of the differentially regulated isoforms of STAT3 in the transgenic tissue is at present unknown.

NF-κB and STAT3 regulate numerous genes involved in inflammation and growth transformation and their persistent activation is observed in many cancers [31]. In this transgenic model, multiple inflammatory chemokines and cytokines were found to be deregulated and of particular note, CD30, a costimulatory molecule belonging to the TNFR family and its ligand CD153 were found to be induced. Several chronic inflammatory disorders, including psoriasis and atopic dermatitis, are associated with increased numbers of mast cells as well as upregulation of CD30 and CD153 [41]. CD30 is also expressed on endothelial cells in a large proportion of neoplastic and reactive vascular lesions [42] including the neoplastic Reed-Sternberg cells of HD and anaplastic large cell lymphoma (ALCL), and high serum levels of CD30 are correlated with poor prognosis in HD patients [43]. Expression of CD30 in normal tissues is limited, making it a good therapeutic target, indeed anti-CD30 treatment has been shown to be efficacious in ALCL [44] and elimination of CD30 was shown to significantly reduce airway inflammation in a model for allergic asthma [45]. CD30 expression by endothelial cells has also been seen in the inflammatory condition of sclerosing angiomatoid nodular transforming (SANT), which can be EBV positive [46]. The ligand, CD153, is overexpressed in a variety of skin inflammations and in the mast cells within HD tumours [47], as well as showing increased levels (along with increased mast cells) in the synovium and serum of rheumatoid arthritis patients [48]. CD30 has been shown to lead to degranulation independent secretion of chemokines such as MIP-1 from mast cells [41]. The high levels of both CD153 and CD30 detected in the transgenic ear tissue, as well as members of the MIP family suggest that this could be one mechanism of release of mast cell factors here. CD30 and CD153 showed considerable upregulation particularly in the later stages (St4 and St5) of the transgenic tissue with no expression detected in controls.

CD30 expression is thought to be regulated in part through the promoter AP1 site and particularly via JunB which is deregulated in several malignancies [49,50]. We have previously shown increased AP1 activity in the transgenic ear tissue and marked upregulation of JunB, which could underlie induction of CD30 in this model [21]. However, it is not clear if these activities are present in the same cellular compartment as the induced CD30 and CD153 expression, with CD153 detected primarily in the vascular endothelial cells and mast cells. Moreover, consistent JunB induction from an early age and phenotypic stage was observed suggesting direct upregulation by LMP1, while CD30 and CD153 induction was detected at the later stages in mice usually older than 4 months, indicating this upregulation follows a cascade of events *in vivo*.

Strong L-selectin staining was seen in the granules of mast cells with a weak staining in the epidermis (both nuclei and cytoplasm). L-selectin is a glycan receptor involved in leukocyte trafficking and implicated in a number of inflammatory disorders. Mast cell precursors are thought to be recruited from the blood, migrating from the bone marrow to the tissue, where they differentiate and mature [51]. L-selectin deficiency has been found to inhibit mast cell recruitment to a repeatedly antigen stimulated skin site [52]. Thus the expression of L-selectin in the mast cells of the transgenic skin may be required for the recruitment of the mast cells to the site and blockade of L-selectin could inhibit this and potentially alleviate the pathology. Recently selectins have become increasingly viable targets in the therapy of inflammatory diseases [53].

IL-3, produced by activated T-cells, monocytes and stromal cells can induce the proliferation, maturation and survival of several hematopoietic cell types, including mast cells [54,55]. It has overlapping functions with GM-CSF and both of these factors were indicated to be increased in the transgenic tissues by array analysis. IL-3 induction in the transgenic tissue was confirmed by western blotting suggesting that the transgenic tissue is supportive for mast cell survival.

CXCL13 (BCL) was notably upregulated in the transgenic tissue samples from the array data. CXCL13 is implicated in the trafficking of B-cells into tissues and has been shown to be upregulated in NPC biopsies, as has CD40 [7], also found upregulated in the transgenic samples. CD40 is a costimulatory protein for antigen presenting cells, particularly B-cells and macrophages, furthermore CD40/CD40L signalling is required for T-cell dependent B-cell differentiation and antibody secretion.

CXCL10 (CRG2 or IP10), a ligand for CXCR3, regulates leukocyte trafficking. It is a chemokine that is associated with tissue damage and necrosis [56] and its over-expression has been observed in several autoimmune and inflammatory conditions, including psoriasis [57,58]. CXCL10 is induced in several cell types by IFNγ (which was detected at increased levels in the transgenic tissue) and in turn attracts Th1 cells to generate a positive feedback loop [59]. CXCL10 was upregulated in the transgenic tissue. Although from the array data induction was seen at both St2 and St5, by western, clear upregulation was only detected in the latter stage.

IL-1 is an important mediator of inflammation acting as an activator of T- and B-cells and NK cells [60]. IL-1 is overexpressed in psoriatic skin [61] and induces hyperplastic epidermal lesions in transgenic mice [62,63], with several similarities to the phenotype observed in our LMP1 transgenic mice. IL-1 is also implicated in other inflammatory disorders such as rheumatoid arthritis, inflammatory bowel disease and atherosclerosis and has been shown to promote auto-antibody production in the murine lupus model MRL/lpr mice [64]. We observed increased levels of IL-1β, but decreased levels of IL-1α in the LMP1 transgenic skin. IL-1 has been shown to stimulate the production of IL-2 but inhibit IL-4 expression, consistent with this, IL-2 was found at higher levels in the transgenic skin samples (>3 fold) while IL-4 was not induced. Furthermore, targets of IL-1 were also found to be induced: GM-CSF was increase 2 fold and S100A9 was previously found to be considerably elevated in a proteomic analysis [33].

IL-8 (or CXCL8) binds to the CXCR1 and CXCR2 receptors on neutrophils, inducing their recruitment and activation and has been detected at elevated levels in a proportion of NPC, HD and BL samples [13,65,66]. All three rodent analogues, CXCL1/KC, CXCL2/MIP2 and CXCL5-6/LIX were observed at higher levels in the transgenic tissue, particularly MIP2 which can recruit both neutrophils and lymphocytes *in vivo *[67].

TGFβ1 is the most potent known neutrophil chemoattractant; large numbers of neutrophils were seen in the stage 4 and 5 tissues in which increased TGFβ1 levels were detected, particularly around the necrotic areas. TGFβ1 induction might also be expected to inhibit the proliferation and activity of mature helper and cyotoxic T-cells as well as NK cells. Also, TGFβ1 augments regulatory type T-cells to dampen immunosurveillance, including NKT cells [68,69]. Additionally, TGFβ1 in conjunction with IL-2 (also found at higher levels in the transgenic tissue as noted above), induces FoxP3^+ ^Treg cells [70], which were found in the transgenic tissue but not in controls. Treg cells secrete IL-10 which was found at higher levels in the transgenic tissue, which would contribute to immunosuppresion [71]. LMP1 has been found to exert immunosuppressive effects [72,73], which would be compounded in the transgenic tissue by the release of TGFβ1. Moreover, the sustained induction of Rae-1 would impair the actions of NK cells in immunosurveillance. Therefore the transgenic tissue environment is one of sustained inflammation with predicted suppression of cytotoxic activites.

Several induced factors in the transgenic skin recruit or activate B- or T-cells and indeed T-cell infiltration was evident as well as a significant IgG deposition. The relevance of these cells in the pathology was demonstrated by their genetic deletion in RAG1 null mice. The inflamed state and degree of hyperplasia of the transgenic tissue was profoundly limited in the absence of B- and T-cells. In the RAG1-null background, the LMP1 induced phenotype remained at a stage of mild hyperplasia (described here as St2), with a failure to recruit the innate immune cells seen in a wild type background. Thus B- and/or T-cells are necessary mediators for the progression of the phenotype. Using an HPV16:E6/E7 transgenic model, de Visser *et al. *[5] demonstrated that soluble B-cell-derived or induced factors were capable of partially restoring the carcinogenic skin phenotype in a B-cell/T-cell (RAG1) deficient background. If the similarity between the models extends to this finding then the IgG deposition noted here may be a critical mediator in the progression of the LMP1 induced phenotype. We hypothesize that the role of B-cells in the carcinogenic progression of this model lies in immunoglobulin production, which is deposited in the tissue. The role of the T-cells is likely to be multifold: in providing B-cell help and in secreting certain cytokines, such as IL-3 which then goes on to induce and support several hematopoietic cell types, including mast cells; but also in modulating the environment through the suppressive activity of Treg cells and their secretion of IL-10 and TGFβ1. The subsequent recruitment of mast cells contributes to the cascade of events leading to chronic inflammation.

Of note, in this analysis we have compared the inflamed, hyperplastic, but pre-neoplastic transgenic tissue with controls; as such, some factors noted to be affected by LMP1 in human tumour samples but not observed here (such as IL-4 and IL-6), could reflect our focus on incipient neoplasia in this study. Also, some observations from our study are likely to be indicative of skin-specific responses, possibly different in type to those seen in the mucosal epithelium of NPC. In particular, observations not noted in EBV-associated disease, but found in common with the transgenic carcinoma model expressing E6 and E7 of human papilloma virus (HPV)-16 in the skin [5], may reflect a tissue-specific programme. In this respect, it is becoming apparent that different tissues or organs initially recruit different immune cell subsets. Recruitment of B-cells or B-cell factors can be a feature of skin, breast and pancreatic tumours [25], while tumours of other organs might preferentially recruit T-cell support at an early phase. Thus (as with the HPV16:E6/E7 model), the relevance of B-cells and/or T-cells to the pathology probably reflects both the actions of the initiating oncogene (LMP1 in this case) as an inducer of proliferation and inflammation, as well as the tissue under study in the model, in this case the skin. It can be hypothesized that, when expressed in a different tissue, LMP1 will lead to inflammatory cell recruitment, but possibly with an altered leukocyte constitution reflecting the tissue type.

## Conclusions

In this model, we have shown that transgenic expression of an oncogene of EBV, LMP1, induces changes in the levels of numerous proteins involved hyperproliferation, oxidative stress, angiogenesis, metastasis and inflammation [20,21,33]. Here we have identified changes in the levels of several key cytokines and chemokines involved in inflammation and shown that the tissues are inflamed. The importance of the B-cell and/or T-cell infiltrate is demonstrated through its elimination, which limits the pathology in these mice to an early stage of benign hyperplasia.

The developing pathology in this model presents a number of points for potential therapeutic intervention. These could be applied where relevant both to LMP1 expressing EBV associated carcinomas, as well as skin tumours and conditions that show a similar pathology. A hypothesis of the sequential events can be proposed as follows: expression of LMP1 in the epidermis leads to the activation of multiple signalling pathways and the deregulation of several causal factors in proliferation, angiogenesis and inflammation; which we observe in the young mice with tissue pathology of St1 and St2. Factors upregulated or activated include EGFR and its ligands, VEGF, AP1, NF-κB [20,21] and STAT3, and the immediate pathology is one of hyperplasia and increased vascularisation. Subsequently, B-cells and T-cells are recruited, possibly through the induction of trafficking factors such as CXCL13 and CXCL10, and immunoglobulins are deposited in the tissue. This leads to mast cell maturation and recruitment, possibly mediated by IL-3 and L-selectin. Deposited IgG and CD30 and CD153 may then promote the release of further factors from the mast cells. The consequences of this cascade of events is the chronically inflamed tissue denoted here as St5, from which neoplastic lesions can arise. Under this hypothesized scheme, CXCL10 and CXCL13, B-cell and Ig deposition, L-selectin and CD30/CD153 [47] could represent candidate target points in the therapy of LMP1-expressing carcinomas, as well as more generally skin carcinomas and certain inflammatory conditions, such as atopic dermatitis showing similar pathological features.

## Methods

### Transgenic mice

L2LMP1^CAO ^(line 117 and line 105B) transgenic mice in a >99% FVB background were used in these studies [20]. The line 117 mice were cross-bred with recombinase activating gene-1 null (RAG1^-/-^) mice maintained in a C57Bl/6 background [74]. F1 LMP1/RAG1^+/- ^males were back-crossed to RAG1^-/- ^females to produce a cohort of offspring with RAG1 heterozygote and null genotypes, thereby ensuring consistency in mouse age, strain and environmental conditions. The ear phenotype was staged 1-5 for a cohort of 43 mice every one to two weeks for ≥180 days. Statistical comparison was made by Kaplain-Meier curve plots of development of phenotypic stage over time. All female mice (LMP1 negative due to Y chromosomal transgene insert) displayed no ear phenotype. All procedures have been conducted under UK Home Office license and the research has complied with Home Office and institutional guidelines and policies. Tissue samples were frozen in liquid N_2 _and stored at -70°C for sample extraction or formalin fixed at 4°C for immunohistochemical analyses. Serum was isolated by allowing blood to clot overnight, centrifuging at 14,000 g for 10min and the supernatant was stored at -70°C.

### Immunohistochemistry

Formalin-fixed paraffin embedded (FFPE) tissues were sectioned at 2 μm for hematoxylin and eosin (H&E) staining and IHC. Washed H&E was used to detect eosinophils. Astra blue (revealing mast cells) stained sections were counter-stained with safranin. Antibodies were investigated against FFPE tissue using a two-step IHC technique. Epitope retrieval was achieved using a microwave pressure cooker (850 W) in 10 mM sodium citrate pH6 buffer. Sections were stained using an EnVision+ system α-rabbit HRP kit (Dakocytomation, K4010) as per manufacturer's instructions. Following staining, all sections were washed in H_2_O, counterstained with Gill's hematoxylin, differentiated in 1% acid alcohol then the nuclei blued in Scott's tap water substitute. IHC antibodies were directed to: CD3 (A0452) used at a dilution of 1:100; myeloperoxidase (A0398) 1:2000, lysozyme (A0099) (Dakocytomation) 1:1000, CD19 (ebioscience) 1:30, von Willebrand factor (Abcam) 1:750, CD153 (Biolgened) 1:500, IL-3 (Santacruz) 1:500, L-selectin (antibodies online) 1:50. Images were captured using a Zeiss Axioskop 2 microscope and KS300i software (imaging associates).

### Isolation of haematopoetic cells from ear tissue and flow cytometry

Ears were collected from line 117 St3 or St4 mice and negative controls (using two to three times as many controls to obtain sufficient cell numbers). Following optimisation (additional file 1 figures S6, S7 and table S3), the tissue was minced with a blade in PBS (2 ml/ear), then incubated in the presenene of collegenase II and collegenase IV (10 mg/ml), 0.5 mg/ml DNase I with 3 mM CaCl_2 _at 37°C for 30 mins. At 30 mins, dispase (final concentration 0.5%) was added and the samples were further incubated for 15 mins. Two volumes DMEM containing 10% FBS were then added and the cells passed through a 30 μm filter (Miltenyi). Cells were washed (194 × g, 5 min) and resuspended at 2.5 × 10^7 ^cells/ml in PBS/1% FBS. Isolated cells for analysis by flow cytometry were pre-incubated by adding goat serum to 10%, for 10 mins, washed and resuspended in PBS/1%FBS. Cells (at least 10^6^/sample) were stained with FITC, PE, PerCP or APC conjugated antibodies (eBioscience) directed to: CD45, CD3, CD4, CD8, NK1.1, for 20 mins at 4°C. 7-AAD was used as a live/dead cell discriminator. Intracellular staining for FoxP3 and Granzyme B was performed according to manufacturers guidelines. Briefly, cells were stained with antibodies against CD4 or CD8, and CD25 (for FoxP3) or CD8 (for Granzyme B). The cells were then fixed by incubating with fixative solution (eBioscience) for 20 mins at 4°C. The cells were washed twice with permeablization buffer (eBioscience) and incubated with anti FoxP3 or anti Granzyme B for 30 mins at 4°C in permeabilization buffer. Finally samples were washed in PBS/1%FBS and analysed using a flow cytometer (BD FACSAria or FACSCalibur) and FlowJo (9.1) software.

### Western blotting

Proteins were extracted in RIPA buffer and were separated (40-200 μg per track) by SDS-PAGE (7.5%, 10% or 15%), with blotting and blot-washing performed as previously described [75]. For probing, the blots were incubated in 5% non-fat milk PBS 0.1% (v/v) Tween 20 with the appropriate anti-sera dilution. Antibodies (with dilutions) used were directed to: TGFβ (Abcam) 1:2000, Rae-1 (R&D Systems) 1:1000, CD30 (Santacruz) 1:1000, phospho-ser727-STAT3 and total-STAT3 (Cell signalling) 1:1000, GAPDH (Santacruz) 1:1000, and antibodies as above under IHC but at a dilution of 1:1000; followed by the appropriate 1:4000 goat anti-rabbit, goat anti-mouse or donkey anti-goat IgG HRP-conjugates (Santacruz). Detection was performed by enhanced chemiluminescence (liteAblot kit, Euroclone).

### Cytokine array [Raybio^R ^Mouse Cytokine Antibody Array 3 (#AAM-CYT-3)]

Serum or tissue protein extracts were pooled and assayed according to the manufacturers protocol. Proteins were extracted from tissues using the supplied lysis buffer, supplemented with 1 mM PMSF and protease inhibitor cocktail (Sigma:100 mM benzamidine, 200 mg/ml leupeptin, 200 mg/ml pepsatin A), on ice for 15 min, clarified by centrifugation (10 min, 4°C 16,000 g) then stored in aliquots at -70°C. After blocking (1hr at room temp.) the membranes were incubated with 1.2 ml of sample (1:10 dilution of serum or 350 μg tissue extract in supplied diluent) at 4°C o/n. The membranes were then washed 3 × 5 min with 2 ml wash buffer I at RT, then 2 × 5 min with 2 ml of wash buffer II at RT. The detection antibody cocktail (biotin-conjugated) was diluted in 2 ml of blocking buffer and 1 ml was added to each of two membranes which were then incubated at 4°C o/n. Following incubation the membranes were washed as before and then 1 ml of HRP-conjugated streptavidin at 1:1000 dilution was added to each membrane and incubated for 2 hrs at RT, then washed as above. Antigens were visualised using the supplied luminol system and signal intensities quantified using MacBAS V2.2 software with scanned images.

## Abbreviations

C1-C5: non-transgenic sibling controls to St1-St5 samples (i.e. age matched); CCB: colloidal Coomassie blue; CXCL13: CXC ligand 13 (or BLC :B-lymphocyte chemoattractant); EBV: Epstein-Barr virus; FFPE: Formalin-fixed paraffin embedded; H&E: hematoxylin and eosin; het: heterozygous; IL: interleukin; LMP1: latent membrane protein 1; LMP1^CAO^: nasopharyngeal carcinoma CAO variant of LMP1; MIP: macrophage inflammatory protein; NK: natural killer; NSC: non-transgenic sibling control; RAG: recombinase activating gene; RT: room temperature; St1-St5: stage 1 to stage 5; TGF: transforming growth factor; WT: wild type

## Competing interests

The authors declare that they have no competing interests.

## Authors' contributions

AH and MAQ contributed equally to the work presented in this manuscript. AH carried out the RAG1 null cross breed studies and the initial identification of inflammatory infiltrates; MAQ conducted the majority of the western blot analyses presented, the flow cytometry experiments and prepared the histology data shown in figures 4 and 6; CN provided the histology service: embedding, sectioning and immunostaining tissues; SJ and PT conducted the cytokine array; AP provided expert histopathological analysis of tissue sections; JBW conceived, coordinated, designed and procured funding for the study and wrote the manuscript. All authors read and approved the final manuscript.

## Supplementary Material

Additional file 1 **Supplementary information**. This file contains tables S1, S2 and S3 along with the optimisation protocol for obtaining cell suspensions from ear tissue, with figures S6 and S7. Click here for file

Additional file 2 **Supplementary information**. This file contains figures S1 to S5. Click here for file
