# Supplementary material for: Lymphocyte deficiency limits Epstein-Barr virus latent membrane protein 1 induced chronic inflammation and carcinogenic pathology in vivo
Source: Mol Cancer. 2011 Feb 3;10:11. doi: 10.1186/1476-4598-10-11 (PMC3041781; doi:10.1186/1476-4598-10-11)
Supplement: Additional file 1 — Supplementary information. This file contains tables S1, S2 and S3 along with the optimisation protocol for obtaining cell suspensions from ear tissue, with figures S6 and S7. [file 1476-4598-10-11-S1.PDF]

Inhibition of EBV latent membrane protein 1 induced chronic inflammation limits the carcinogenic pathology *in vivo*

Adele Hannigan, M. Asif Qureshi, Colin Nixon, Penelope Tsimbouri, Sarah Jones, Adrian Philbey and Joanna B. Wilson

Additional File 1 supplementary information

Table S1 The categorised stages of the L2.LMP1^{CAO} line 117 ear phenotype

Ear stage	Mouse age	Phenotypic description
1	up to ~5 weeks	Increasing vascularisation with acanthosis.
2	1-2 months	Clear hyperplasia, with acanthosis and increased vasculature.
3	2-5 months	Acanthosis, hyperkeratosis, parakeratosis, increased vasclature and evidence of inflammation.
4	4-8 months	Severe hyperplasia including parakeratosis and hyperkeratosis, erosive or ulcerative dermatitis, necrosis, with fibrovascular hyperplasia of underlying dermis.
5	≥ 5 months	As stage 4, with increasing necrosis, developing keratoacanthoma (or papilloma) and occasional carcinoma.

LMP1 is expressed at the highest levels in the skin of the ear of the L2LMP1^{CAO} mice and this is reflected by the observation that this tissue shows the greatest phenotype. The mouse age indicates the usual age range for the phenotype of the mouse ear to reach the stage indicated for the line 117 in the FVB strain background (which is shown in Stevenson *et al*., (2005) Cancer Research **65**:8826-8835). The L2LMP1^{CAO} line 105B expresses lower levels of LMP1 and the phenotype is slower to develop than in line 117, although follows the same pattern. While the phenotype has been categorised into recognisable stages for experimental convenience, this reflects a continuum rather than discrete phases of the advancing pathology. Keratoacanthoma, papilloma and carcinoma present as discrete lesions.

<u>Table S2</u> Comparison of cytokine and chemokine levels between transgenic and control ear tissue and serum samples

A] Cytokines and chemokine levels higher in transgenic samples compared to NSC

Serum St5/C5		Tissue St2/C2		Tissue St5/C5	
**cytokine**	**ratio**	**cytokine**	**ratio**	**cytokine**	**ratio**
CD30	16.49	CD30L	∞	CD30L	∞
TIMP-1	9.01	CD30	∞	CD30	∞
MIP-1γ	6.06	CXCL13(BLC)	18.06	CXCL13(BLC)	18.42
CD30L	4.17	CXCL10(CRG-2)	11.86	CXCL10(CRG-2)	11.47
Leptin	3.20	CD40	8.80	MIP-3α	7.09
IL-3Rβ	2.62	L-Selectin	8.50	CD40	5.21
Eotaxin	2.48	IL-3	6.11	IL-12P40/P70	5.17
FasLigand	2.27	MIP-2	4.20	IL-3	4.60
CXCL10(CRG-2)	2.19	MIP-3β	3.92	L-Selectin	4.19
RANTES	2.06	sTNF RII	3.83	MIP-3β	3.23
CTACK	2.05	IL-12P40/P70	3.76	IL-2	3.10
IL-1β	1.99	IL-3 Rβ	3.73	MIP-2	2.84
CXCL16	1.88	LIX	3.72	CXCL16	2.70
SCF	1.70	IL-2	3.39	AXL	2.61
IL-2	1.70	MIP-3α	3.30	IL-3 Rβ	2.30
TNFα	1.69	TIMP-1	3.14	GCSF	2.28
TECK	1.60	IL-1 β	3.09	VEGF	2.25
L-Selectin	1.54	IGFBP-5	2.95	LIX	2.23
Eotaxin-2	1.49	TPO	2.85	IFNγ	2.19
IL-1α	1.49	IL-13	2.71	GM-CSF	2.18
IL-3	1.37	IL-17	2.69	IL-1 β	2.16
IL-5	1.35	GCSF	2.42	IGFBP-5	1.93
Fractalkine	1.31	VEGF	2.41	Lymphotactin	1.89
MIP-2	1.18	VCAM-1	2.30	sTNF RII	1.87
VEGF	1.10	PF-4	2.19	CTACK	1.86
CXCL13(BLC)	1.10	KC	2.11	IL-10	1.77
M-CSF	1.10	AXL	2.10	Fractalkine	1.67
VCAM-1	1.04	IL-10	2.10	TPO	1.61
P-Selectin	1.03	Leptin R	2.05	TIMP-1	1.57
IL-6	1.00	GM-CSF	2.04	MIG	1.53
		IFNγ	2.00	P-Selectin	1.52
		Leptin	1.95	PF-4	1.45
		CXCL16	1.93	Leptin	1.38
		Lymphotactin	1.83	Leptin R	1.36
		P-Selectin	1.69	MIP-1α	1.35
		MIP-1α	1.66	IL-13	1.34
		CTACK	1.61	Eotaxin	1.32
		TNFα	1.51	KC	1.29
		IGFBP-3	1.50	Fas Ligand	1.24
		RANTES	1.43	VCAM-1	1.23
		MIG	1.40	IL-5	1.23
		IL-12P70	1.19	RANTES	1.18
		SCF	1.14	IL-12P70	1.17
		SDF-1α	1.13	IGFBP-3	1.15
		sTNF RI	1.12	sTNF RI	1.11
		MIP-1γ	1.05	TNFα	1.09
				MIP-1γ	1.07
				IL-17	1.05
				SCF	1.03
				SDF-1α	1.01

B] Cytokines and chemokine levels lower in transgenic samples compared to NSC

Serum St5/C5		Tissue St2/C2		Tissue St5/C5	
**cytokine**	**ratio**	cytokine	ratio	cytokine	ratio
IL-6	1.00	Eotaxin	0.88	IL4	0.97
LeptinR	0.97	IL-6	0.88	IL-6	0.97
IL-4	0.95	MCP1	0.88	M-CSF	0.93
MG	0.92	IL-5	0.87	MCP1	0.93
PF-4	0.91	M-CSF	0.87	MCP-5	0.67
sTNFRI	0.84	IL4	0.84	IL-9	0.63
MIP-1α	0.81	Fractalkine	0.76	eotaxin2	0.61
sTNFRII	0.77	TECK	0.74	TECK	0.56
GM-CSF	0.74	IL-1α	0.63	TCA-3	0.56
GCSF	0.72	Fas Ligand	0.61	TARC	0.52
LIX	0.71	IL-9	0.59	IL-1α	0.38
Lymphotactin	0.70	TCA-3	0.57	IGFBP-6	0.36
IL-9	0.65	MCP-5	0.55		
IGFBP-6	0.62	TARC	0.53		
MCP1	0.62	IGFBP-6	0.37		
TPO	0.62	eotaxin2	0.31		
MCP-5	0.60				
SDF-1α	0.57				
IL-12p70	0.57				
TCA-3	0.56				
Axl	0.54				
IL-10	0.54				
TARC	0.50				
IGFBP-3	0.48				
MIP-3β	0.46				
KC	0.46				
IL-12p40/p70	0.46				
IL-13	0.43				
MIP-3α	0.35				
IL-17	0.25				
IFNγ	0.23				
IGFBP-5	0.20				

A pool of four samples (from different mice) were used for each test sample assayed by cytokine immunodetection array. Samples were taken from serum from transgenic mice with St5 ear phenotype and serum from non-transgenic sibling controls (NSC) (1st column), transgenic stage 2 ear tissue extracts (St2) compared with C2 (2nd column) and transgenic stage 5 ear tissue extracts (St5) compared with C5 (3rd column). The mean of the normalised values obtained for each cytokine was taken and the ratio of transgenic over NSC determined. All those with a ratio ≥1 are shown in table [A] and with a ratio ≤1 in table [B]. Those showing a two fold or greater difference between the transgenic sample compared to NSC are shaded. ∞= indeterminate ratio as none detected in control while readily detected in transgenic sample

Optimisation of a single cell suspension from mouse ears and for T-cell characterisation
In the first instance, a trypsin/collagenase protocol was assessed, varying the concentration of the enzymes and digestions times. Pairs of ears from control mice were minced manually by blade and incubated at 37^oC, rotating in 1x trypsin plus 2.5 mgml^{-1} collagenases types II and IV (Gibco) and 0.5 mgml^{-1} DNase I (Sigma) for 30 min. In order to aid the activities of the collagenases, 3 mM $CaCl_2$ was then added for 15 min. The digestion was quenched via the addition of two volumes of DMEM supplemented with 10% FBS and the cells were filtered using a 30 μm diameter filter. This method yielded only 7500 cells per ear, which was insufficient for further analysis.

Next, mouse ears were minced manually and incubated at 37^oC, rotating in 5 x trypsin, 2.5 mgml^{-1} types II and IV collagenases, 0.5 mgml^{-1} DNase I for one hour. This method yielded 1.85 x 10^5 and 9.50 x 10^4 cells per ear for the ears minced without or with collagenase respectively.

Next, gentle homogenisation of the mouse ears was used instead of manual mincing, in 5 x trypsin and incubated, rotating, 37^oC for 45 min. 3mM $CaCl_2$ was then added to one of the samples for a further 15 min whilst a second continued incubation in the absence of $CaCl_2$. The sample incubated in the presence of $CaCl_2$ yielded an increased number of cells per ear in comparison to the ear extract incubated in the absence of $CaCl_2$ (4 x 10^5 and 2.45 x 10^5 cells per ear respectively).

	Trypsin	Collagenases
**Treatment 1**	5 x	2.5 mgml^{-1}
**Treatment 2**	5 x	5 mgml^{-1}
**Treatment 3**	5 x	10 mgml^{-1}
**Treatment 4**	10 x	5 mg ml^{-1}

Table S3 Ears were homogenised into either 5 x or 10 x trypsin as indicated above and incubated in the presence of varying concentrations of collagenases types II and IV.

Subsequently, cells were homogenised in the presence of either 5 x trypsin (Table S3, treatments 1-3) or 10 x trypsin (Table S3, treatment 4) and subjected to enzymatic digestion in the presence of 0.5 mgml^{-1} DNase I and 2.5, 5 or 10 mgml^{-1} collagenases types II and IV (table S3). All treatments were performed in duplicate and trypsin digestion was for either 30 min or 45 min followed by a 30 min or 15 min incubation in the presence of collagenases, 0.5 mgml^{-1} DNase I and 3mM $CaCl_2$. In all cases, the total incubation time was 1 h. Following filtration, it was found that treatment 3 gave the highest yield of cells (2.2 x 10^5 cells per ear, figure S6) when the homogenised tissue was incubated at 37^oC, rotating in the presence of 5 x trypsin for 45 min prior to incubation with 10 mgml^{-1} collagenases types II and IV and 0.5mgml^{-1} DNase I for 15 min. This protocol was then used for isolation of kerainocytes for analysis,

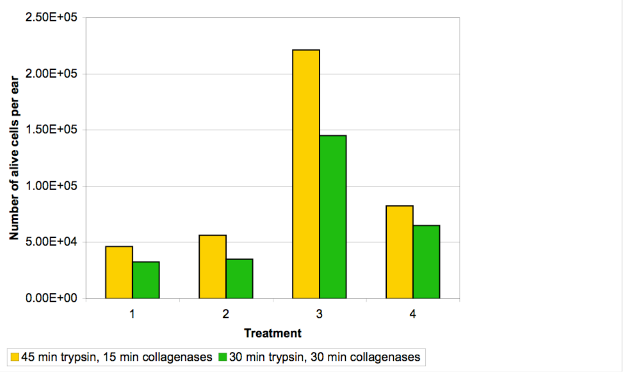

Figure S6 Number of cells extracted per ear following incubation with varying concentrations of trypsin and collagenases types II and IV. Green bars indicate samples incubated for 30 min in the presence of trypsin and 30 min in the presence of collagenases II and IV. Yellow bars indicate samples incubated for 45 min in the presence of trypsin and 15 min in the presence of collagenases II and IV.

Trypsin treatment and prolonged dispase treatment can reduce T-cell surface marker detection. Therefore a further modified protocol was assessed for leukocyte isolation. Pairs of ears were taken from transgenic mice and detection of CD45, CD4 and CD8 markers used to assess quality. Ears were either minced manually with a blade or gently homogenised in 2ml/ear PBS. Samples were then incubated in the presence of 10mgml^{-1} collagenase II and

collegenase IV, 0.5 $mgml^{-1}$ DNase I and 3mM $CaCl_2$ at 37°C for 30 mins. At 30 mins, dispase was added to a final concentration of 0.5% to some samples and all samples were incubated for a further 15 minutes. Two volumes DMEM containing 10% FBS were then added and the cells passed through a 30 µm filter (Miltenyi). Cells were washed (194xg, 5 min) and resuspended in PBS, 1% FBS. Isolated cells for analysis by flow cytometry (usually $5x10^6$ cells/200µl) were pre-incubated for 10 mins by adding 20µl of goat serum/200µl sample, then washed and resuspended in 200µl PBS, 1%FBS. Cells were stained with FITC, PE, PerCP or APC conjugated antibodies (eBioscience) directed to: CD45, CD4 and/or CD8 for 20 min at 4°C, in the first instance for optimisation (figure S7). 7-AAD was used as a live/dead cell discriminator. Samples were washed in PBS, 1%FBS and analysed using a flowcytometer (BD FACSAria or FACSCalibur) and FlowJo (9.1) software.

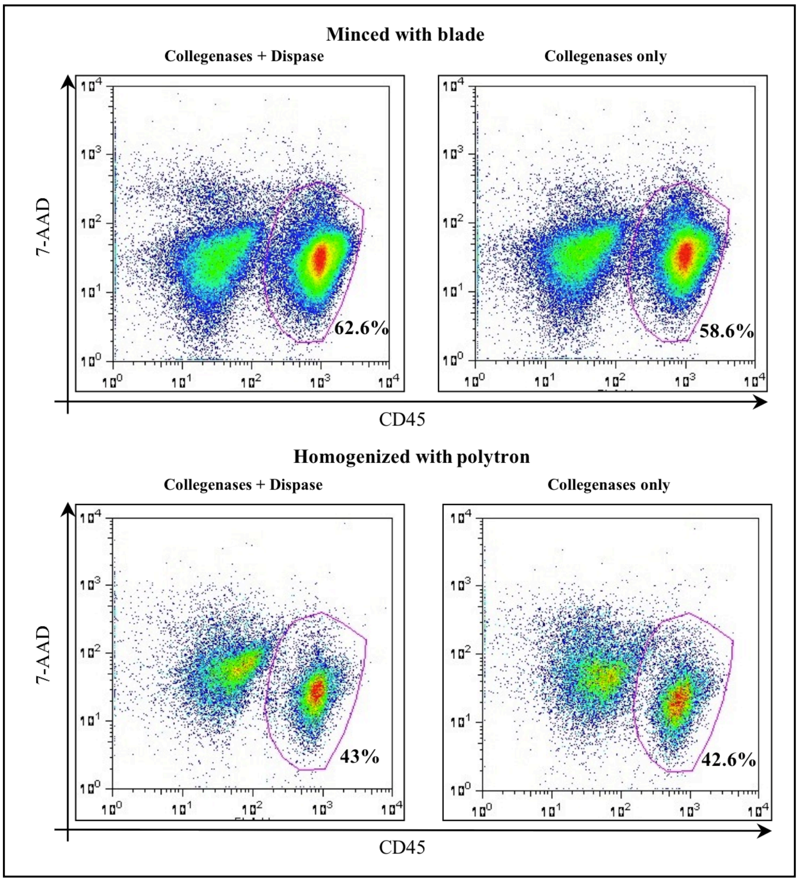

<u>Figure S7</u> Staining of viable leukocytes following collagenase with or without dispase cell isolation. Cells were isolated by mincing the tissue or gentle homogenisation followed by treatment with collagenases, plus or minus brief dispase treatment, followed by detection of 7AAD and CD45 by flow cytometry, using $2x10^5$ cells per flow sample (the plots shown have been pre-gated on the live cell population by FSC and SSC).

Mincing the tissue finely followed by treatment with collagenases and brief dispase treatment yielded the greatest number of live leukocytes (62.6%) and equivalent CD4/CD8 staining as treatments without dispase (not shown). In the absence of trypsin, this cell isolation might be expected to release fewer keratinocytes than the treatment described above, but permits T-cell surface marker evaluation.
