# Supplementary material for: Lymphocyte deficiency limits Epstein-Barr virus latent membrane protein 1 induced chronic inflammation and carcinogenic pathology in vivo
Source: Mol Cancer. 2011 Feb 3;10:11. doi: 10.1186/1476-4598-10-11 (PMC3041781; doi:10.1186/1476-4598-10-11)
Supplement: Additional file 2 — Supplementary information. This file contains figures S1 to S5. [file 1476-4598-10-11-S2.PDF]

Inhibition of EBV latent membrane protein 1 induced chronic inflammation limits the carcinogenic pathology *in vivo*

Adele Hannigan, M. Asif Qureshi, Colin Nixon, Penelope Tsimbouri, Sarah Jones, Adrian Philby and Joanna B. Wilson

Additional file 2: Supplementary Information

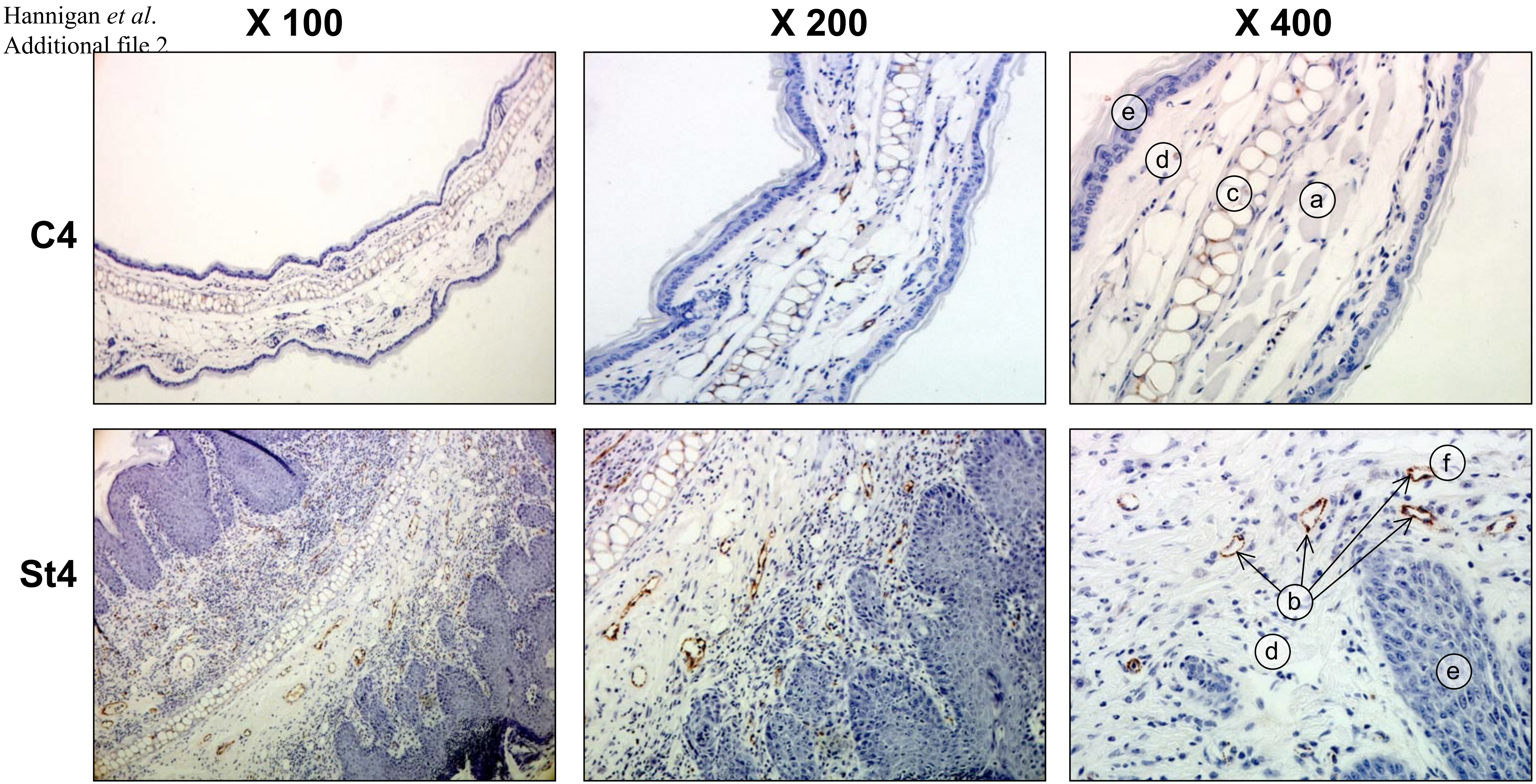

Figure S1: Sections of St4 ears and age matched controls (C4) were immunostained (brown) with antibodies to von Willebrand factor (vWF) as a marker for blood vessels and angiogenesis. Original magnification (x100, x200, x400) indicated. Example cells/structures are indicated:
(a) adipose tissue
(b) blood vessel
(c) cartilage
(d) dermis
(e) epidermis

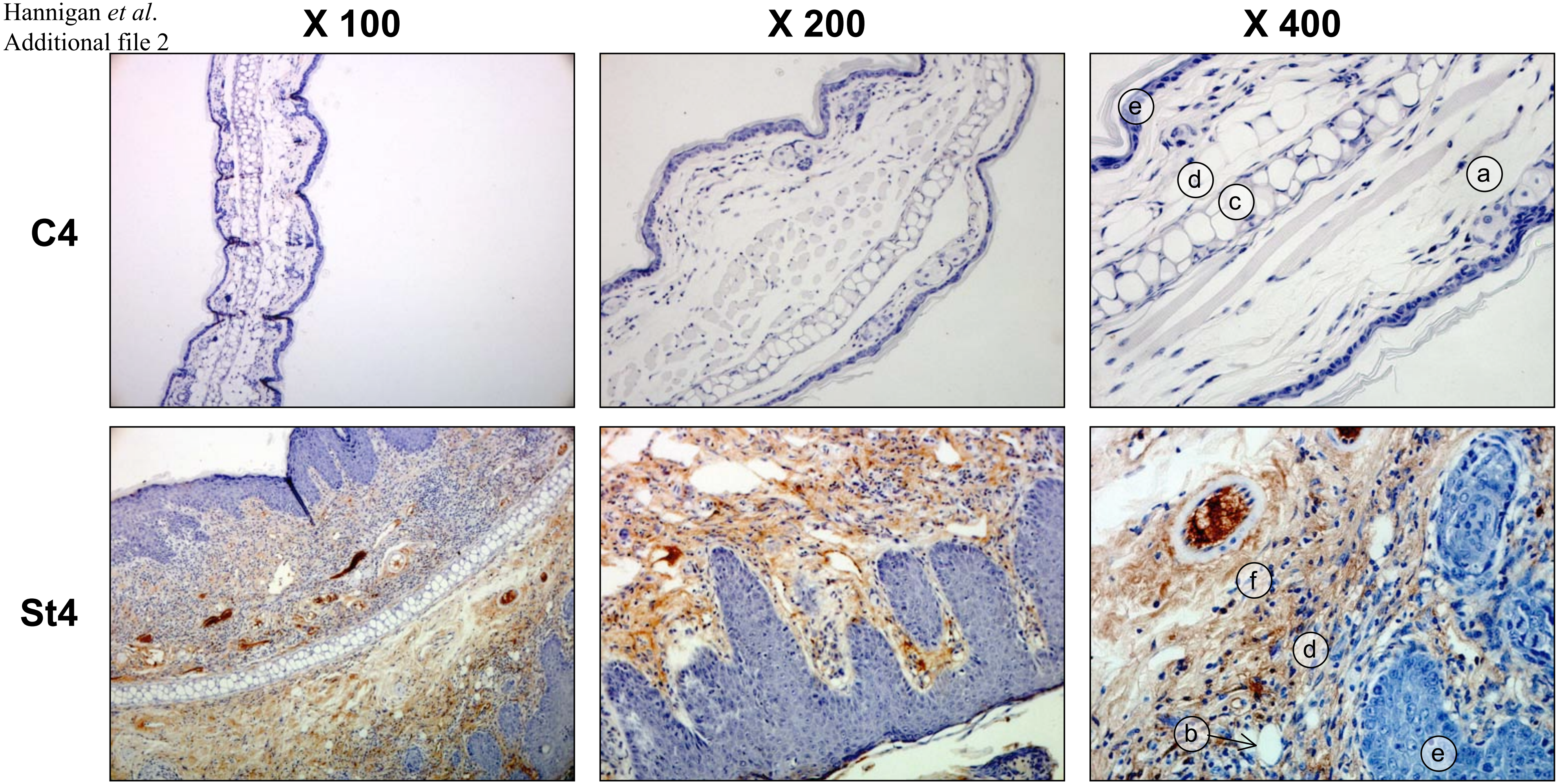

Figure S2: Sections of St4 ears and age matched controls (C4) were immunostained (brown) with antibodies to IgG. Original magnification (x100, x200, x400) indicated. Example cells/structures are indicated:
(a) artifactual split in tissue
(b) blood vessel
(c) cartilage
(d) dermis
(e) epidermis
(f) positive staining in dermis

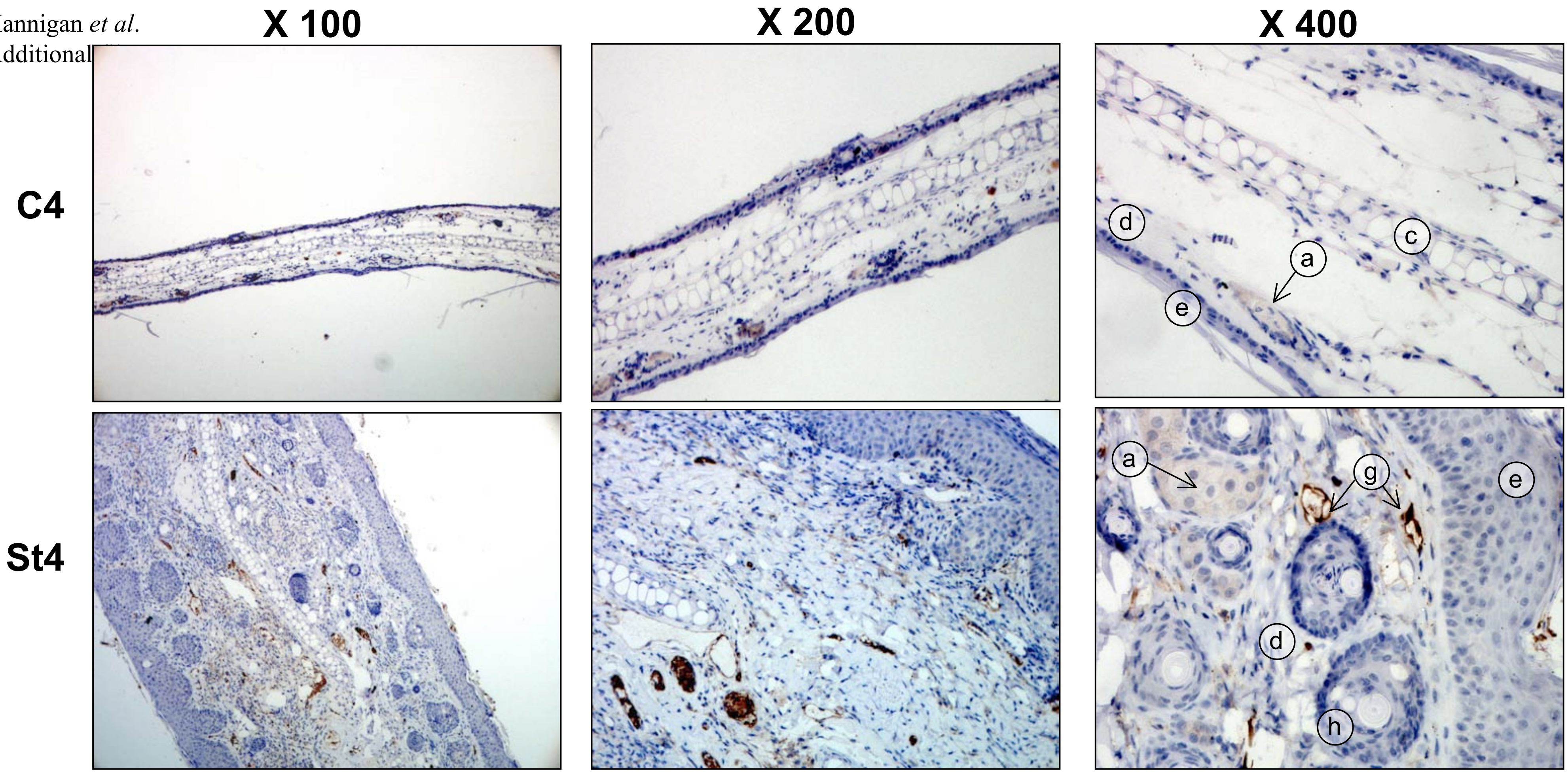

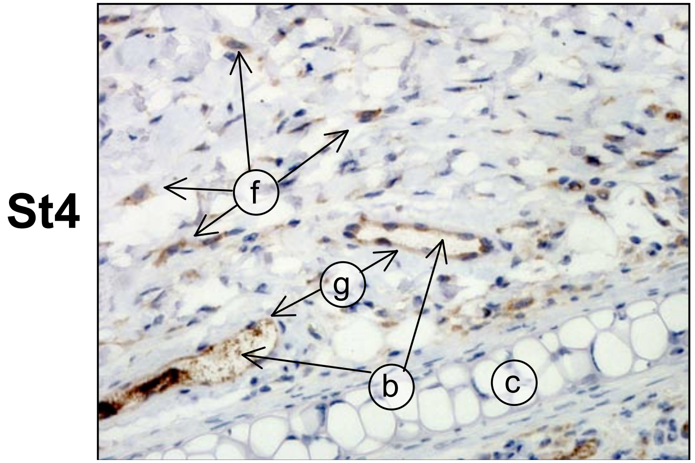

Figure S3: Sections of St4 ears and age matched controls (C4)were immunostained (brown) with antibodies to CD153/CD30L. Original magnification (x100, x200, x400) indicated.
Examples cells/structures are indicated:

(a) sebaceous gland with non-specific staining
(b) blood vessel
(c) cartilage
(d) dermis
(e) epidermis
(f) positive staining in fibroblasts and/or mast cells
(g) positive staining in vascular endothelial cells
(h) hair follicle

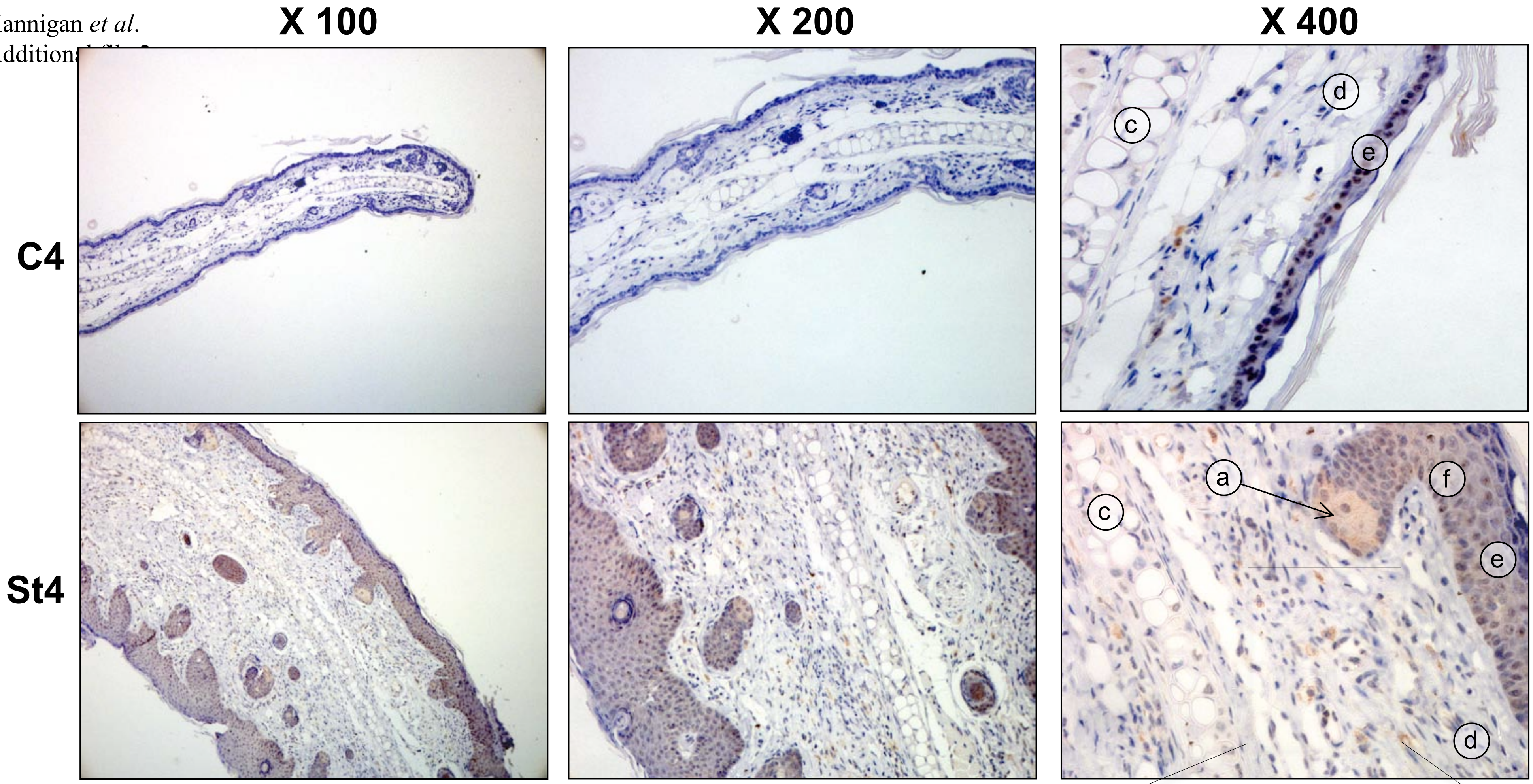

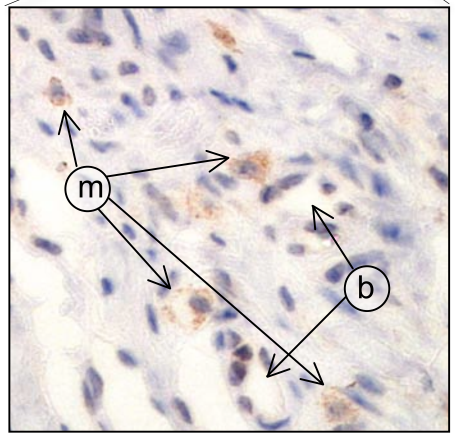

Figure S4: Sections of St4 ears and age matched controls (C4) were immunostained (brown) with antibodies to L-selectin. Original magnification (x100,x200, x400) indicated. Examples cells/structures are indicated:
(a) sebaceous gland with non-specific staining
(b) blood vessel
(c) cartilage
(d) dermis
(e) epidermis
(f) weak positive staining in nuclei and cytoplasm of epidermal cells
(m) granular positive staining in cytoplasm of mast cells

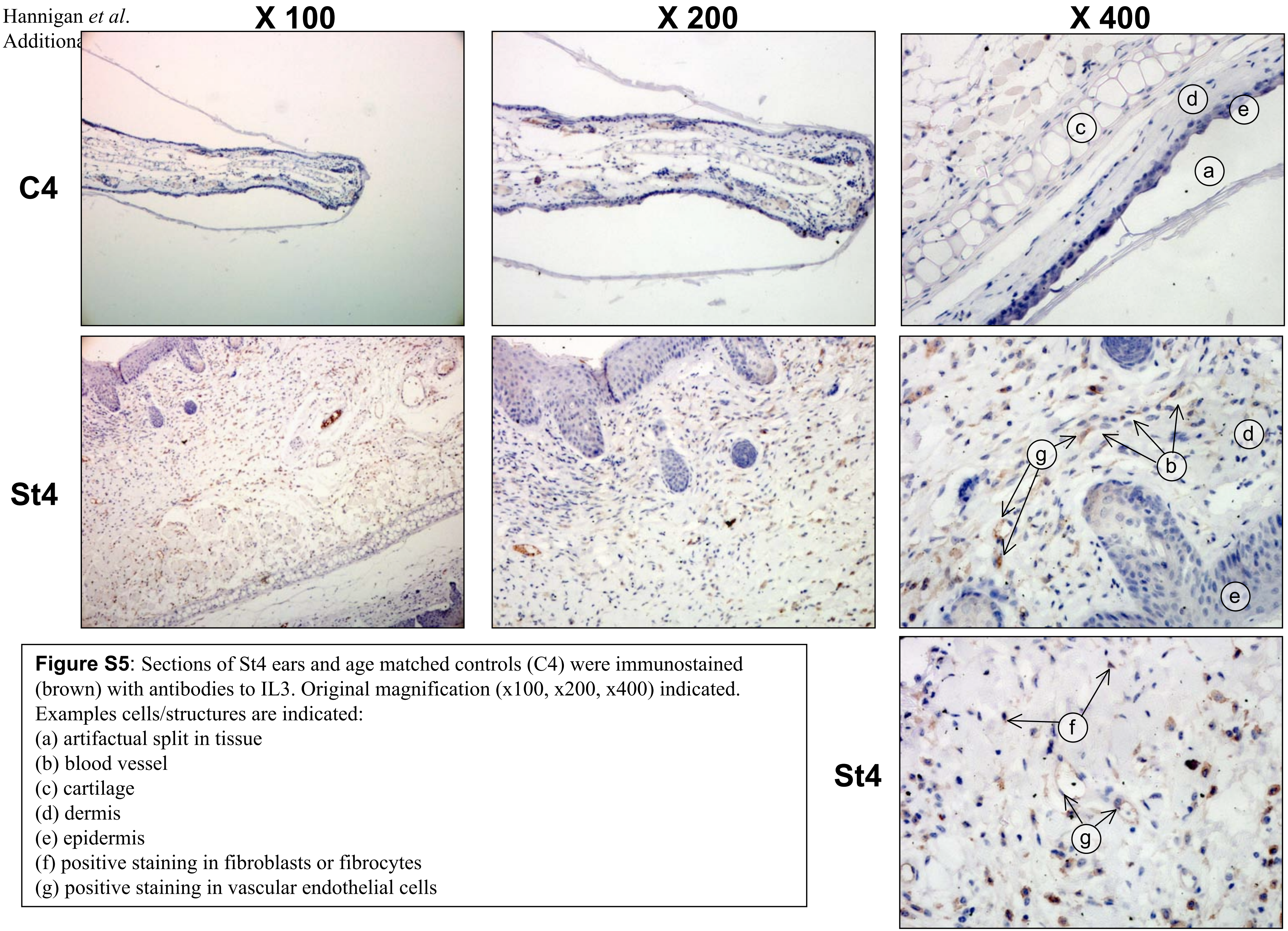

Figure S5: Sections of St4 ears and age matched controls (C4) were immunostained (brown) with antibodies to IL3. Original magnification (x100, x200, x400) indicated. Examples cells/structures are indicated:

(a) artifactual split in tissue
(b) blood vessel
(c) cartilage
(d) dermis
(e) epidermis
(f) positive staining in fibroblasts or fibrocytes
(g) positive staining in vascular endothelial cells
